# Enhancing Podocyte Degenerative Changes Identification With Pathologist Collaboration: Implications for Improved Diagnosis in Kidney Diseases

**DOI:** 10.1109/JTEHM.2024.3455941

**Published:** 2024-09-10

**Authors:** George Oliveira Barros, José Nathan Andrade Muller da Silva, Henrique Machado de Sousa Proença, Stanley Almeida Araújo, David Campos Wanderley, Luciano Rebouças de Oliveira, Washington Luis Conrado Dos-Santos, Angelo Amancio Duarte, Flavio de Barros Vidal

**Affiliations:** Instituto Federal Goiano 185121 Goiania 76300-000 Brazil; Department of PathologyFederal University of Bahia 28111 Salvador 40110-909 Brazil; Department of PathologyKidney and Hypertension Hospital, Oswaldo Ramos Foundation SÃo Paulo 04038-002 Brazil; Center for Electron Microscopy, Institute of NephropathologyFederal University of Minas Gerais 28114 Belo Horizonte 31270-901 Brazil; Intelligent Vision Research Laboratory, Institute of ComputingFederal University of Bahia 28111 Salvador 40170-115 Brazil; Gonçalo Moniz Institute, Oswaldo Cruz Foundation Salvador 40296-710 Brazil; Department of TechnologyState University of Feira de Santana 67836 Feira de Santana 44036-900 Brazil; Department of Computer ScienceUniversity of Brasilia 28127 Brasília 70910-900 Brazil

**Keywords:** Computational nephropathology, podocyte degenerative changes, glomeruli, deep learning, decision-making.

## Abstract

Podocyte degenerative changes are common in various kidney diseases, and their accurate identification is crucial for pathologists to diagnose and treat such conditions. However, this can be a difficult task, and previous attempts to automate the identification of podocytes have not been entirely successful. To address this issue, this study proposes a novel approach that combines pathologists’ expertise with an automated classifier to enhance the identification of podocytopathies. The study involved building a new dataset of renal glomeruli images, some with and others without podocyte degenerative changes, and developing a convolutional neural network (CNN) based classifier. The results showed that our automated classifier achieved an impressive 90.9% f-score. When the pathologists used as an auxiliary tool to classify a second set of images, the medical group’s average performance increased significantly, from $91.4\pm 12.5$% to $96.1\pm 2.9$% of f-score. Fleiss’ kappa agreement among the pathologists also increased from 0.59 to 0.83. Conclusion: These findings suggest that automating this task can bring benefits for pathologists to correctly identify images of glomeruli with podocyte degeneration, leading to improved individual accuracy while raising agreement in diagnosing podocytopathies. This approach could have significant implications for the diagnosis and treatment of kidney diseases. Clinical impact: The approach presented in this study has the potential to enhance the accuracy of medical diagnoses for detecting podocyte abnormalities in glomeruli, which serve as biomarkers for various glomerular diseases.

## I. Introduction

Podocyte lesions can be caused by genetic predisposition or by kidney injury resulting from infection, toxicity, hemodynamics, or obesity. These lesions may be severe, resulting in podocyte histological changes such as cell swelling, vacuolization, cell detachment of the glomerular basement membrane, binucleation, and proliferation. Severe podocyte lesions correlate with nephrotic level proteinuria, or nephrotic syndrome [1], and can be present in collapsing nephropathy, focal segmental glomerulosclerosis, lupus nephropathy [2], diabetic nephropathy (DN) [3], [4] and immunoglobulin A nephropathy (IgAN) [5].

Severe podocyte lesions may also induce genetic program in tubular cells that provokes tubulointerstitial inflammation, fibrosis, and tubular atrophy or favors the development of focal and segmental glomerular sclerosis, contributing to progressive loss of renal function [4], [5]. Although reversible slight podocyte lesions can usually be only perceived by electron microscopy analysis, severe podocyte degeneration can be diagnosed through the histological study of renal glomeruli on light microscopy. In recent years, several automated, machine learning-based methods have been proposed to aid in the visual analysis of renal tissue slides [6], [7], [8], [9]. When writing this manuscript, the literature contained no references to computational models specifically focused on the automated classification of podocyte degenerative changes using images of renal glomeruli.

Some authors segmented podocytes for quantitative studies in segmental sclerosis, diabetic nephropathy, and anti-neutrophil antibody-associated glomerulonephritis [10], [11], [12], [13], [14], [15]. Murazesk et al. [13] analyzed podocyte morphological changes and depletion by segmenting them in PAS-stained images. On the other hand, Barros et al. [15] used attribute extraction and convolutional neural networks (CNN) to classify glomeruli images as having or lacking morphological changes in podocytes based on a small dataset with low image diversity.

Although the reports mentioned above aimed to automate the segmentation of podocytes in glomeruli images, this task is not directly useful for making medical decisions about the presence or absence of podocyte degeneration. Instead, classifications are more relevant for this purpose. Moreover, previous studies did not focus on datasets that specifically included podocyte alterations diagnosed by pathologists using light microscopy analysis. Therefore, the models trained on these datasets were not reliably prepared to segment podocytes within glomeruli. Additionally, these datasets lacked diversity in terms of staining techniques. They did not represent real-world images well and did not include other lesions commonly associated with the disease under study. As a result, these datasets provided limited examples compared to those encountered in the routine practice of pathologists.

Motivated by the advances of applying CNN in the field of histology [16], [17] and by the desire to provide the clinical translational aspect, this study aims to fill gaps in computational pathology research on podocytes in renal glomeruli. To this end, we developed an automated binary classification algorithm based on CNN to classify glomeruli images according to the presence of podocyte degeneration. We compared the performance of this classifier to that of three pathologists who evaluated the same images under three different scenarios. In the first scenario, we assessed the performance of the automated classifier alone. In the second scenario, three pathologists visually analyzed the glomerular images and determined whether podocyte degeneration was present or absent using a standard method. Finally, the pathologists reevaluated the images based on the classifications established by the automated algorithm. We compared the results of each scenario with the level of proteinuria (the nosological diagnosis) related to each image.

As an additional contribution, we created a new dataset of glomeruli images with podocyte degenerative lesions. This dataset consists of 1,143 samples stained with four different techniques (PAS, H&E, PAMS, and trichome). It features a variety of lesions (sclerotic, membranous, and hypercellular) to represent the complexity of images encountered by pathologists in real-world practice. This diverse dataset enhances the reliability of trained models, as they were applied to cases where podocytopathy appeared under different image acquisition conditions and in the context of associated diseases. Finally, this new dataset may also encourage further research on podocytopathy identification.

## II. Material and Methods

The experiments were executed in three steps, following a protocol inspired by the work of Ligabue et al. [18], which evaluated the performance of a CNN in classifying immune deposits using immunofluorescence images of renal biopsies. The authors then compared the performance of the CNN against that of a group of three trained pathologists.

**FIGURE 1.**
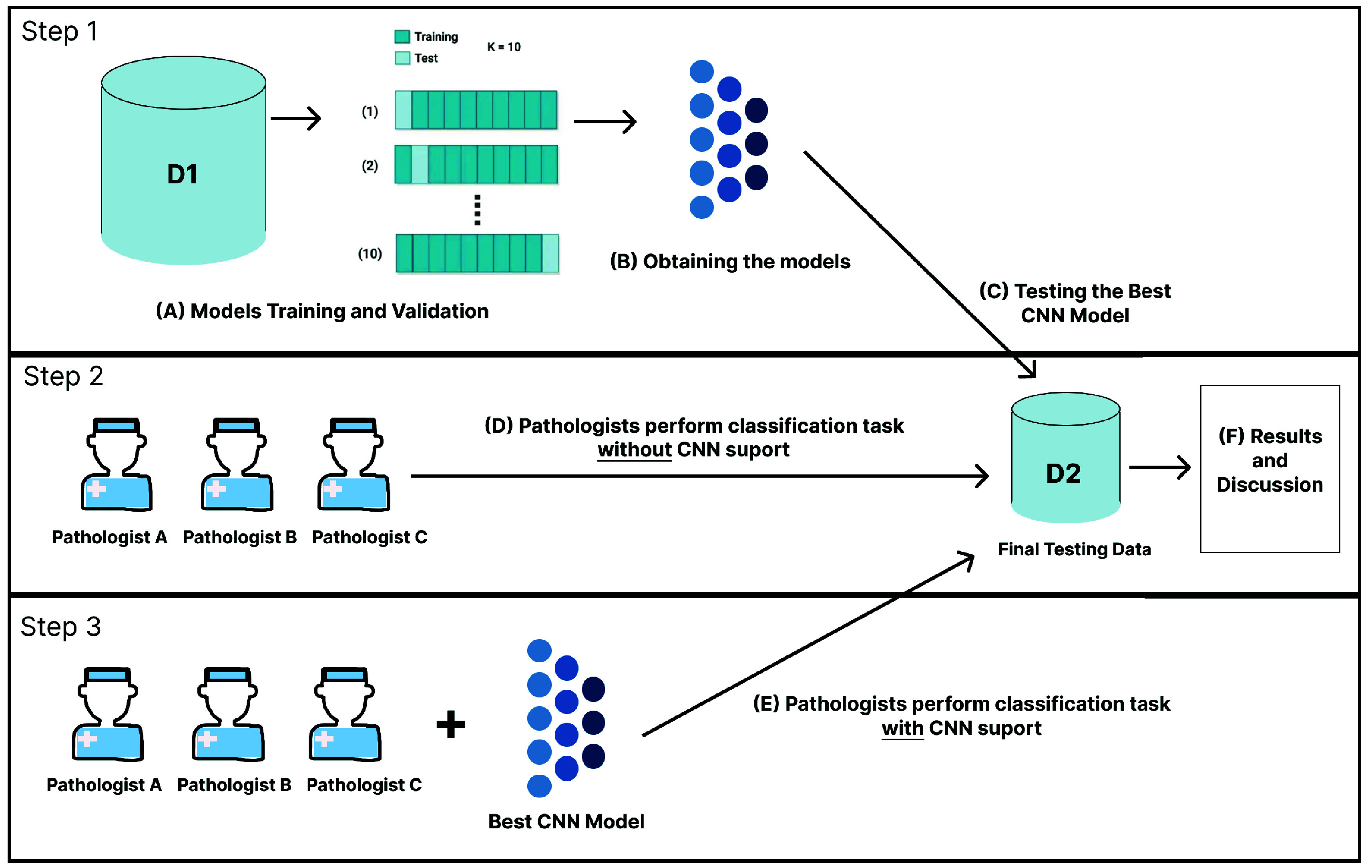
Overview of the conducted experiment protocol. In Step 1, several models based on CNN architecture were evaluated to select the one offering the best performance using dataset D2. In Step 2, we assessed three pathologists’ performance in classifying the images in dataset D2. In Step 3, we evaluated the performance of the same pathologists in classifying the images in dataset D2 with the aid of the results obtained from the automated classifier selected in Step 1.

For the first step, the goal was to obtain an automated classifier capable of recognizing glomeruli with podocyte degeneration visible by light microscopy. Several automated classifiers (based on CNN architecture) were fitted (trained and validated) using dataset D1. The performance of each model in classifying the images in dataset D2 was ranked to select the most efficient model. In the second step, we assessed the performance of a group of three pathologists who classified the images in dataset D2, to compare their performance against that of the selected computational model. Finally, after a period of 30 days had elapsed (a time interval adopted to avoid classification bias), the same group of pathologists evaluated the images in D2 again, this time with the results from the automated classifier to assess whether this information would influence (i.e., improve) the pathologists’ performance. Figure 1 presents an overview of the experiments performed.

### A. Image Data

**FIGURE 2.**
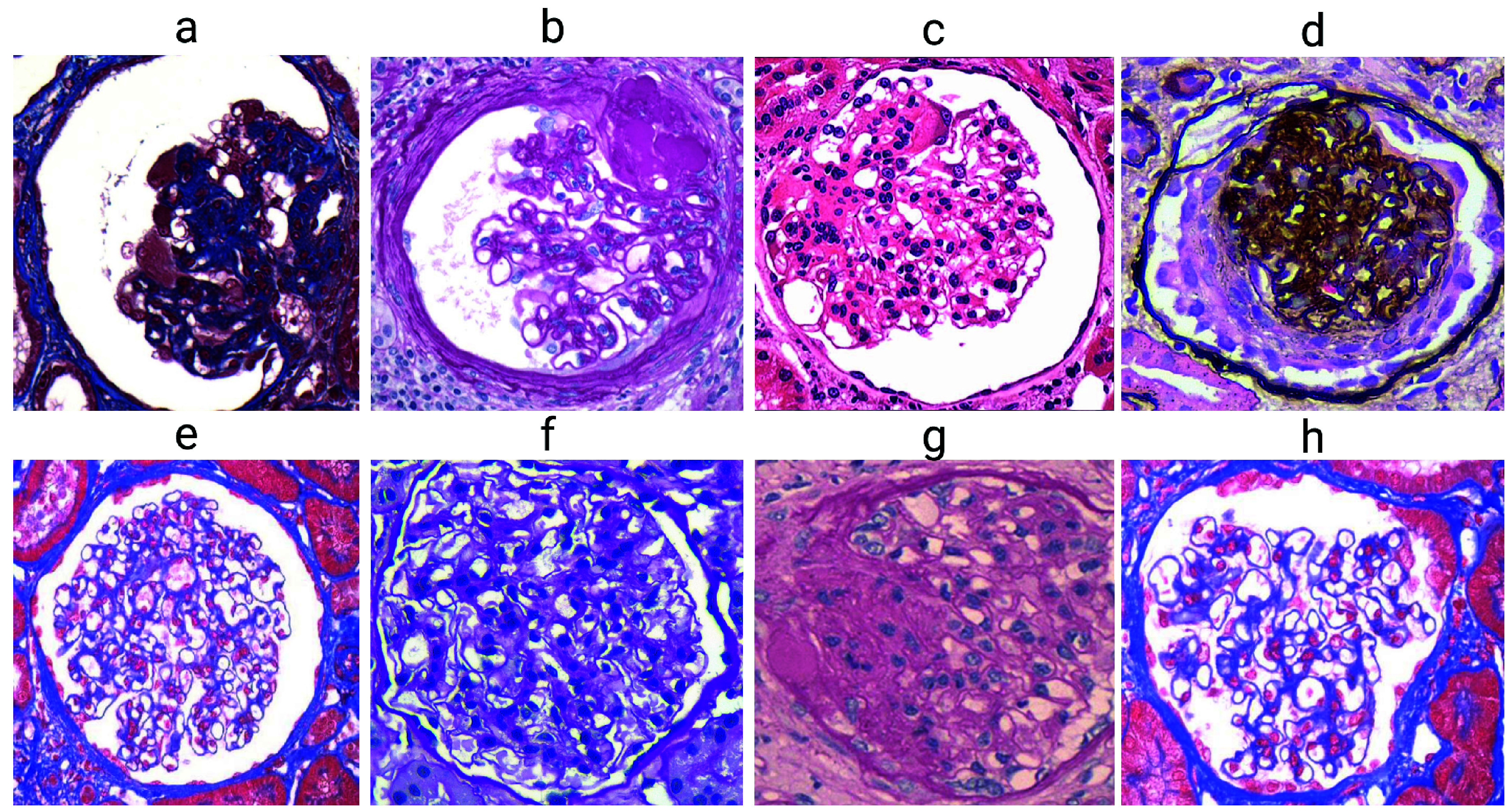
Samples from Dataset 1 (D1). Glomeruli with podocyte degeneration (a-d) and glomeruli without podocyte degeneration (e-h).

We assembled two datasets containing digital images of renal glomeruli. Dataset 1 (D1) was used to train and test the automated classifier, while Dataset 2 (D2) was used for performance assessments. Figure 2 illustrates some examples of the images that comprise datasets D1 and D2. The datasets (D1 and D2) include images of glomeruli with various types of podocyte injuries, such as degeneration, hyperplasia, and hypertrophy. This diversity of podocyte injuries provides a realistic representation of the types of cases encountered in clinical practice, allowing the trained computational models to perform in a manner that reflects the complexity of real-world scenarios. These images did not undergo any preprocessing operations and were delivered to the classifiers exactly as the pathologists provided them. This approach enabled end-to-end processing, as proposed by Goodfellow and colleagues [19].

The images from D1 were obtained by searching image banks provided by two independent pathologists. More than 30,000 images were analyzed, with only 1,043 images selected (513 with and 530 without podocyte degeneration); the selected images employed four different types of staining: PAS, H&E, PAMS, and trichrome (see stain distribution in Table 1). The images originated from the laboratories of different institutions, captured at resolutions ranging from $238\times 201$ to $1920\times 1440$ pixels. In both groups of images (with and without podocyte degeneration), glomeruli contained other unidentified lesions and hypercellular, membranous, and sclerotic lesions labeled by pathologists. The visual inspection of the two independent pathologists who labeled the images in D1 was considered a gold standard used for training the CNN model. Importantly, these two pathologists were not part of the group of three pathologists who performed the classification of dataset D2.

**TABLE 1:** Stain Distribution in Dataset D1 According to Glomeruli With and Without Podocyte Degeneration

Label	H&E	PAS	Trichome	PAMS	Total
With	44	275	104	90	513 (49%)
Without	64	254	94	118	530 (51%)
Total	108 (10.8%)	529 (50.8%)	198 (18.9%)	208 (19.9%)	1043 (100%)

The evaluation dataset (Dataset D2) was created using a different protocol. It consists of 100 images of renal glomeruli (50 with and 50 without podocyte degeneration) stained with PAS, H&E, PAMS, and trichrome. In contrast to Dataset D1, which was labeled solely based on visual inspection by two pathologists, each image in D2 underwent visual inspection and was annotated with proteinuria data as a gold standard. Furthermore, each image in D2 was obtained from a different patient (100 patients) to ensure robustness in the classification results obtained from computational models. This approach also increased the diversity of cases used to evaluate the CNN model’s performance. The distribution of stains in Dataset D2 is shown in Table 2.

**TABLE 2:** Stain Distribution in Dataset D2 according to Glomeruli With and Without Podocyte Degeneration

Label	H&E	PAS	Trichome	PAMS	Total
With	18	3	2	17	50 (50%)
Without	12	24	6	8	50 (51%)
Total	30 (30%)	37 (37%)	8 (8%)	25 (25%)	100 (100%)

### B. Automated Classification

The final automated classifier was developed by comparing six different models, and the one that performed the best was selected. Python 3.6 [20] was used for software coding, along with Tensor Flow 2.6 [21], Numpy 1.21.0 [22], Scikit-learn [23], and KerasTuner [24] libraries. The experiments were run on a desktop with an RTX 2080TI GPU, 64 GB of RAM, and an AMD Ryzen 3 CPU, all running on Ubuntu 22.04 OS.

Three classical CNN [25] architectures were considered for automated image classification: Inception Resnet-v2 [26], Densenet201 [27], and Efficient-Net B3 [28]. These architectures were chosen due to the provision of reliable results in similar tasks [29], [30], [31] and due to differences in architecture concerning depth (the number of layers is fixed in Densenet201 and Inception Resnet-v2, yet variable in Efficient Net B3) and learning strategies (*e.g.*, residual blocks in Resnet, and dense layers in Densenet201). The three network architectures were trained under two scenarios: A) from scratch (FS) with random weight initialization, and B) using transfer learning (TL) where each network was pre-trained with the ImageNet [32] dataset. This combination resulted in six network models to evaluate.

We fine-tuned the hyperparameters for each CNN model using the hyperband [33], [34] algorithm, which incorporates a Bayesian optimization strategy to expedite the search process through adaptive resource allocation and early stopping techniques prior to training.

To adjust the hyperparameters, in D1, the images were randomly divided into two groups: 70% for training and 30% for testing, a common split percentage for training and validating machine learning models. This split maintained the proportion of images in each class, distinguishing between those with and without podocyte degeneration. This split was exclusively employed for adjusting the hyperparameters of each network. This method, as opposed to a conventional random selection of hyperparameters, yielded an enhanced set, contributing to the mitigation of underfitting and overfitting issues.

The hyperband algorithm fine-tuned critical hyperparameters, such as learning rate, optimizer, batch size, loss function, and the number of neurons in the final layers (dense layers) of the network. Table 3 outlines the evaluated values for each hyperparameter. Table 4 shows the results of the best hyperparameter values found for each model candidate.

**TABLE 3:** Range of Values Considered During Hyperparameter Tuning

Hyperparameters	Assessed values
Batch size	32, 16, 8, 4 and 2
Loss function	Binary crossentropy and Hinge
Optimizer	SGD, Adam, Adagrad and RMSprop
Learning rate	0.1, 0.001, 0.0001 and 0.00001
Number of neurons in dense layers	2048, 1024, 512 and 256

**TABLE 4:** Best Hyperparameter Values of Each Model

Model	Bs	Loss	Optimizer	Dense layers	LR
Inception Resnet101 v2 TL	8	Binary Cross-entropy	RMSprop	1024 and 512	0.0001
Densenet201 TL	8	Binary Cross-entropy	Adam	512 and 512	0.001
EfficientNet B3 TL	4	Hinge	RMSprop	Adam and 512	0.0001
Densenet201 FS	4	Binary Cross-entropy	SGD	2024 and 1024	0.001
Inception Resnet 101 v2 FS	8	Binary Cross-entropy	Adam	1024 and 512	0.0001
EfficientNet B3 FS	8	Binary Cross-entropy	Adam	512 and 512	0.0001

After obtaining the best combination of hyperparameters offered by the Hyperband algorithm, as depicted in Figure 1, the training of candidate models involved a 10-fold cross-validation. This process included dividing the D1 set into 10 validation subsets. As a result, each of the 6 network combinations underwent training and validation across 10 iterations, utilizing one subset for validation and the remaining subsets for training in each iteration. Consequently, after these iterations, the cross-validation process yielded 10 distinct models. From these models, the one exhibiting the highest F-score was chosen as the optimal model. Subsequently, the chosen model underwent a comparison with the other optimal models in each of the six combinations.

Continuing our efforts to address the challenges of overfitting and underfitting, and with the goal of augmenting sample diversity throughout the model training process, we integrated a data augmentation strategy into the training sets marked in the cross-validation. This was achieved through the implementation of the following operations: 8 rotations at angles ranging from 30 to 310 degrees (at 30-degree intervals), both vertical and horizontal flips, random adjustments of brightness, and contrast variation. These operations were carefully selected to expand the dataset while avoiding any mischaracterization of the morphology of glomeruli images, which could potentially hinder network learning and compromise the overall classifier performance.

As a result of these augmentation techniques, the training sets for each cross-validation round expanded significantly from 1043 to 12516 images, with each original image generating 12 new images. This augmentation process contributes to a more robust and diverse training dataset, fostering improved generalization capabilities of the classifier.

We adopted an early stop strategy to limit training time, in which training was interrupted whenever a sequence of ten epochs did not reduce loss. This is a common practice also designed to prevent overfitting and unnecessary training time. Finally, after cross-validation and obtaining the 6 best models, the D2 data set was used to test each model.

### C. Pathologist Classification

To establish a baseline for CNN model performance comparisons, three pathologists classified the images in D2 as either “with” or “without” podocyte degeneration. This task was performed exclusively by visually inspecting images without any additional information. Importantly, the D2 classification task was carried out by three pathologists who did not participate in preparing or annotating either of the two datasets considered herein (D1 and D2). Each of the three pathologists is also affiliated with a different nephropathology institution.

To mitigate potential classification bias due to immediate re-analysis, a 30-day washout period was implemented before the same three pathologists re-classified the same images using the output from the automated classifier as additional information. In other words, before deciding on a given image, each pathologist was allowed to consult the results obtained from the automated classifier. This approach allowed us to assess the pathologists’ performance both with and without the support of the computational model, minimizing the risk of over-reliance on the model output.

## III. Results and Discussion

The metrics used to assess the automated classifier and pathologists’ performance were: accuracy, precision, recall, F1-score, and area under the ROC (AUC) curve [35]. In addition to these metrics, we also calculated the reliability of agreement between the three pathologists using the Fleiss’ Kappa [36] metric, precisely to determine whether the inclusion of results from the automated classifier in the image classification task altered inter-observer agreement.

Table 5 lists the average values the CNN models achieve. The model that produced the highest F1-score (96.9%) obtained through 10-fold cross-validation was Inception Resnet101 v2 with transfer learning architecture, offering an overall average of 94.7% of accuracy, 95.5% of precision, 92.3% of recall, and 93.7% of F1-score values. We selected the best one with 96.9% of the F1-score from the ten models obtained by cross-validation.

**TABLE 5:** Results of k-Fold Cross-Validation Indicated the Inception Resnet101 v2 as the Best CNN Model

Model	Acc.(%)	Prec.(%)	Rec.(%)	F1(%)
Inception Resnet101 v2 TL	$94.7\pm 2.6$	$95.5\pm 3.6$	$92.3\pm 4.3$	$93.7\pm 2.6$
Densenet201 TL	$91.3\pm 3.5$	$92.5\pm 3.8$	$90.2\pm 7.1$	$91.1\pm 3.9$
EfficientNet B3 TL	$91.1\pm 2.4$	$92.8\pm 3.4$	$89.0\pm 7.1$	$90.6\pm 2.8$
Densenet201 FS	$88.4\pm 2.9$	$90.6\pm 5.9$	$86.0\pm 5.8$	$88.0\pm 3.1$
Inception Resnet 101 v2 FS	$86.6\pm 3.9$	$89.3\pm 6.0$	$82.2\pm 9.7$	$85.1\pm 4.9$
EfficientNet B3 FS	$83.3\pm 3.1$	$84.1\pm 5.2$	$82.2\pm 7.1$	$82.8\pm 3.4$

**FIGURE 3.**
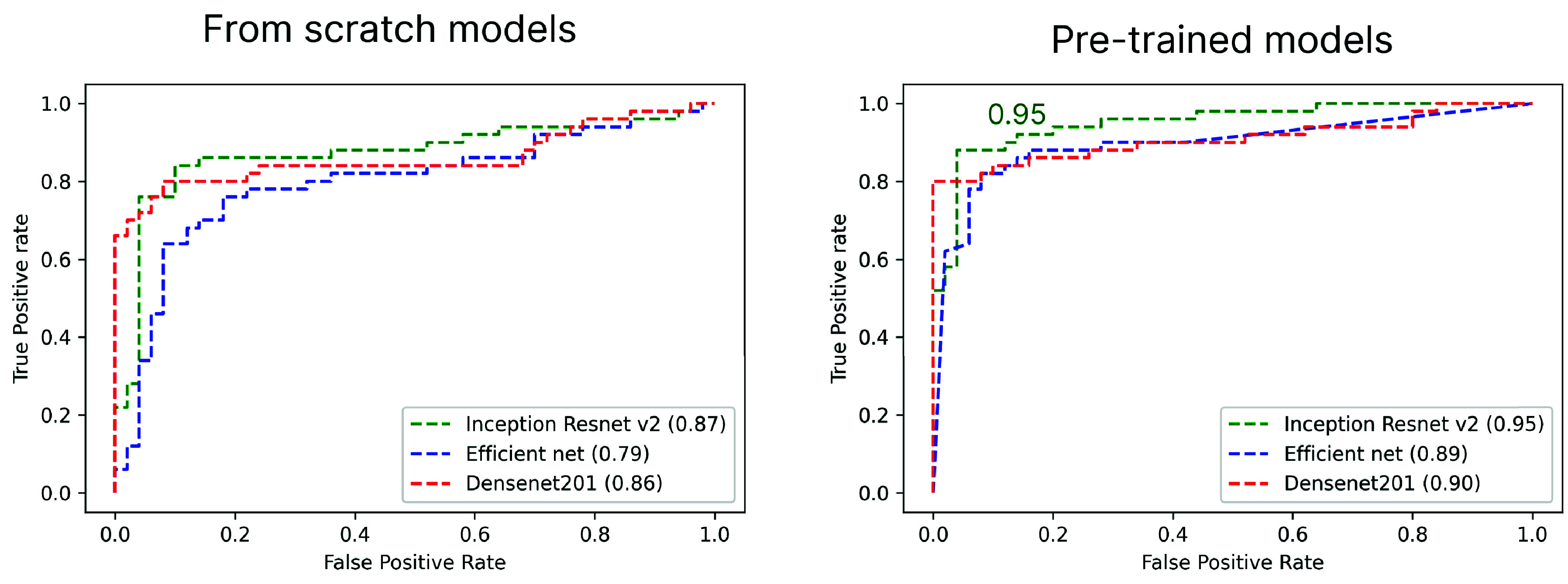
Area under the curve (AUC) of the models when classifying images in the D2 dataset. The figure on the left shows a comparison between the models trained with random weight initialization (“from scratch models”) and the same models trained using transfer learning (“pre-trained models”). The pre-trained models presented a greater AUC in all cases, with the Inception ResNet v2 network offering the best results (0.95, green dotted line).

ROC curves were employed to illustrate the performance of each classifier in conformity with the variability of the discrimination threshold. Ideal/perfect performance is considered when a true positive rate of 1 and a false positive rate of 0 are achieved. Figure 3 shows the ROC curves generated by each model in classifying the images contained in D2. Again, the pre-trained Inception Resnet 101 v2 offered the best discrimination using this dataset, providing an AUC of 0.95 compared to 0.90 from the pre-trained Densenet201. All pre-trained networks surpassed the results obtained by their respective FS versions, which indicates improved performance obtained from using pre-trained networks to classify images of glomeruli with podocyte degenerative changes.

The pathologists’ performance improved when they consulted the results from the automated classifier before providing their classification determinations. Table 6 reveals that the success rate of each pathologist improved independently when using the information obtained from the automated classifier. A comparison of the pathologists’ performance before and after consulting the automated classifier revealed that overall accuracy increased from $86.3~\pm ~10$% to $95.0~\pm ~2$%.

**TABLE 6:** Performance of Three Pathologists With and Without the Aid of an Automated Classifier in Classifying Images From Dataset D2. The Availability of CNN Model Results Showed an Increase In Overall Classification Accuracy

Classifier	Acc.(%)	Prec.(%)	Rec.(%)	F1(%)	Fleiss’ kappa
Pathologist A	94.0	94.0	94.1	94.0	0.59
Pathologist B	91.0	87.0	96.0	91.0	
Pathologist C	74.0	80.0	64.0	90.6	
Pathologist average	$88.3~\pm 10.7$	$87.0~\pm 7.0$	$84.6~\pm 17.9$	$91.4~\pm 12.5$	-
CNN	91.0	86.0	95.0	90.9	-
Pathologist A + CNN	96.0	100.0	92.5	96.1	0.83
Pathologist B + CNN	97.0	94.0	100	96.9	
Pathologist C + CNN	92.0	86.0	97.7	91.4	
Pathologist + CNN average	$95.0~\pm 2.6$	$93.3~\pm 7.0$	$96.7~\pm 3.7$	$96.1~\pm 2.9$	-

Considering the pathologists’ performance in images with and without podocyte degeneration separately, the average accuracy in analyzing images with podocyte degeneration improved from $84.6\pm 17$% to $96.6\pm 3$%. In contrast, the average accuracy of images without podocyte degeneration improved from $88.0\pm 5$% to $98.3~\pm ~2$%. Regarding the pathologists’ performance considering images grouped by staining technique: the overall accuracy for PAS-stained images improved from $83.7~\pm ~9$% to $94.5~\pm ~2$%; for H&E-stained images, from $80.0~\pm ~17$% to $95.5~\pm ~5$%; yet for trichrome-stained images, no difference was observed as average accuracy remained at $91.6~\pm ~7$%; for PAMS-stained images, a discrete decrease was observed, from $96.0~\pm ~6$% to $94.6~\pm ~4$% (within the range of standard deviation).

The automated classifier also contributed to an increase in the degree of agreement (Fleiss’ kappa) between pathologists, which rose from 0.59 (moderate agreement) in their initial analysis to 0.83 (much better agreement) after introducing the aid of the automated classifier – a significant improvement of approximately 40%. Although Fleiss’ kappa is a metric based on Cohen’s Kappa, it differs in that it assesses agreement between three or more observers. This coefficient was calculated to evaluate whether an automated classifier also influences pathologists’ agreement when visually analyzing images.

The increased concordance observed among the three pathologists is particularly noteworthy, given that they are affiliated with different centers. This finding is significant because research reports [37], [38], [39] have shown that pathologists working in the same center tend to exhibit higher agreement rates. The use of pathologists from different centers allowed us to more rigorously evaluate the efficacy of the automated classifier as a support tool.

**FIGURE 4.**
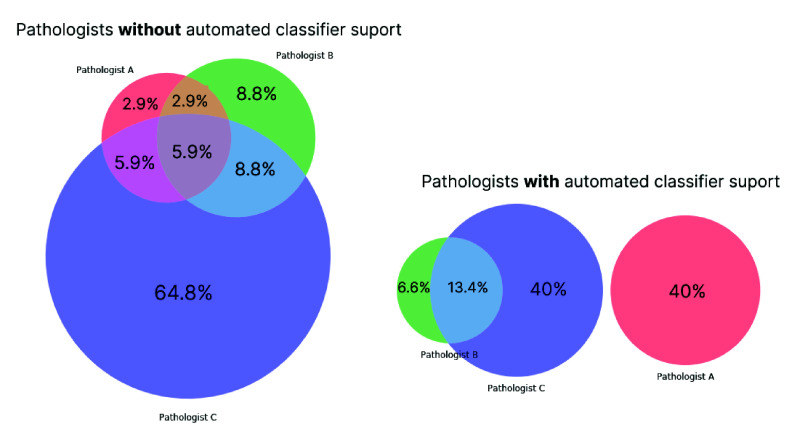
Venn diagrams illustrate the distribution of images misdiagnosed by pathologists with and without an automated classifier. Broader distribution and greater disagreement were observed (seven groups in different colors on the left diagram) without the aid of the CNN model.

Figure 4 presents two Venn diagrams, which illustrate the distribution of errors made by pathologists in their analyses with and without the aid of the automated classifier. It is evident that, following the introduction of the classifier as a support tool, the sets of images that the pathologists misdiagnosed were different. For example, Pathologists A and C committed errors on entirely different sets of images, indicating a clear change in how the pathologists analyzed the images using the information provided by the classifier.

## IV. Conclusion and Future Direction

This study introduces a novel “human-in-the-loop” approach that integrates a convolutional neural network (CNN) with expert pathologist input to accurately identify glomerular podocytopathy. Our findings demonstrate the potential of AI models to augment pathologists’ capabilities in visual analysis of glomeruli with podocyte degeneration. To further contribute to the field, we are making the datasets utilized in this study publicly accessible. By sharing these curated data, we aim to accelerate the development of robust computational models for classifying podocyte degenerative changes in renal glomeruli images.

The limited quantity of training images and the involvement of a relatively small number of pathologists in the annotation process represent significant constraints on the generalizability and robustness of our current models. We acknowledge these limitations and plan to address them in future work by expanding the dataset and involving a larger cohort of pathologists.

We expect that pathologists will be able to adopt automated classifier tools to support the task of image analysis. In our future work, we plan to expand and improve the existing research by incorporating additional datasets to build a multi-class tool capable of performing semantic segmentation of podocytes in glomeruli. This expanded tool will identify and locate podocytes and indicate the specific type of affecting lesion, including normal, degenerative, hyperplastic, or hypertrophic. This comprehensive analysis will provide a more nuanced understanding of podocyte degenerative changes and aid in accurate diagnosis.

Furthermore, we intend to explore and evaluate custom models and other network architectures that may further enhance the performance of the presently developed automated classifier. By investigating alternative deep learning architectures, we aim to improve the accuracy and efficiency of classifying podocyte lesions on conventional slides. This is particularly important as human eyes can only diagnose certain podocyte lesions under electron microscopy, which is time-consuming and resource-intensive. By leveraging advanced network architectures, we anticipate bridging this diagnostic gap and enabling more efficient and accurate diagnosis of podocyte lesions.

Moreover, we recognize the importance of collaboration and data sharing in advancing the field of podocyte degenerative change recognition. Therefore, we plan to collaborate with other research institutions and experts to expand our dataset and ensure a diverse and representative collection of glomerular images. This collaborative effort will facilitate the development of robust and generalizable automated classifier tools that can be applied across different clinical settings and populations.

## Code and Data Availability

Network training scripts and the best podocyte degenerative changes recognition CNN model will be freely available at GitHub repository. Datasets D1 and D2 are available only to researchers and students for academic purposes. Access can be requested through the Pathospotter research group website (http://pathospotter.uefs.br/) or by the following link.

## Author Contributions Statement

George Oliveira Barros performed all deep neural network experimentation. Angelo Amancio Duarte, Luciano Rebouças de Oliveira, Washington Luis Conrado dos-Santos, and Flavio de Barros Vidal reviewed and validated the experimental protocol and experimental results. Washington Luis Conrado dos-Santos and David Campos Wanderley provided images of renal glomeruli, the ground truth for D1 and D2 datasets, and technical support in the medical field. Henrique Machado de Sousa Proença, José da Silva, and Stanley Almeida Araújo performed the classification of the validation dataset (D2). All authors contributed to the writing and revision of the final manuscript.

## Declaration of Competing Interest

The authors declare that they have no known competing financial interests or personal relationships that could have appeared to influence the work reported in this paper.

## Ethical Considerations

The study was conducted in accordance with resolution No. 466/12 of the National Health Council. The patients were not directly involved in the study. The consent was obtained from all subjects and/or their legal guardians, and to preserve confidentiality, the images (including those shown in the paper) were separated from the other patient data. No data presented herein allows patient identification. All the procedures were approved by the Ethics Committee for Research Involving Human Subjects of the Gonçalo Moniz Institute from the Oswald Cruz Foundation (CPqGM/FIOCRUZ), Protocols No. 188/09 and No. 1.817.574.

## Supplementary Materials

Supplementary materials
