# Supplementary material for: Enhancing Podocyte Degenerative Changes Identification With Pathologist Collaboration: Implications for Improved Diagnosis in Kidney Diseases
Source: IEEE J Transl Eng Health Med. 2024 Sep 10;12:635–42. doi: 10.1109/JTEHM.2024.3455941 (PMC11515860; doi:10.1109/JTEHM.2024.3455941)
Supplement: Supplementary materials [file supp1-3455941.pptx]

## Slide 1
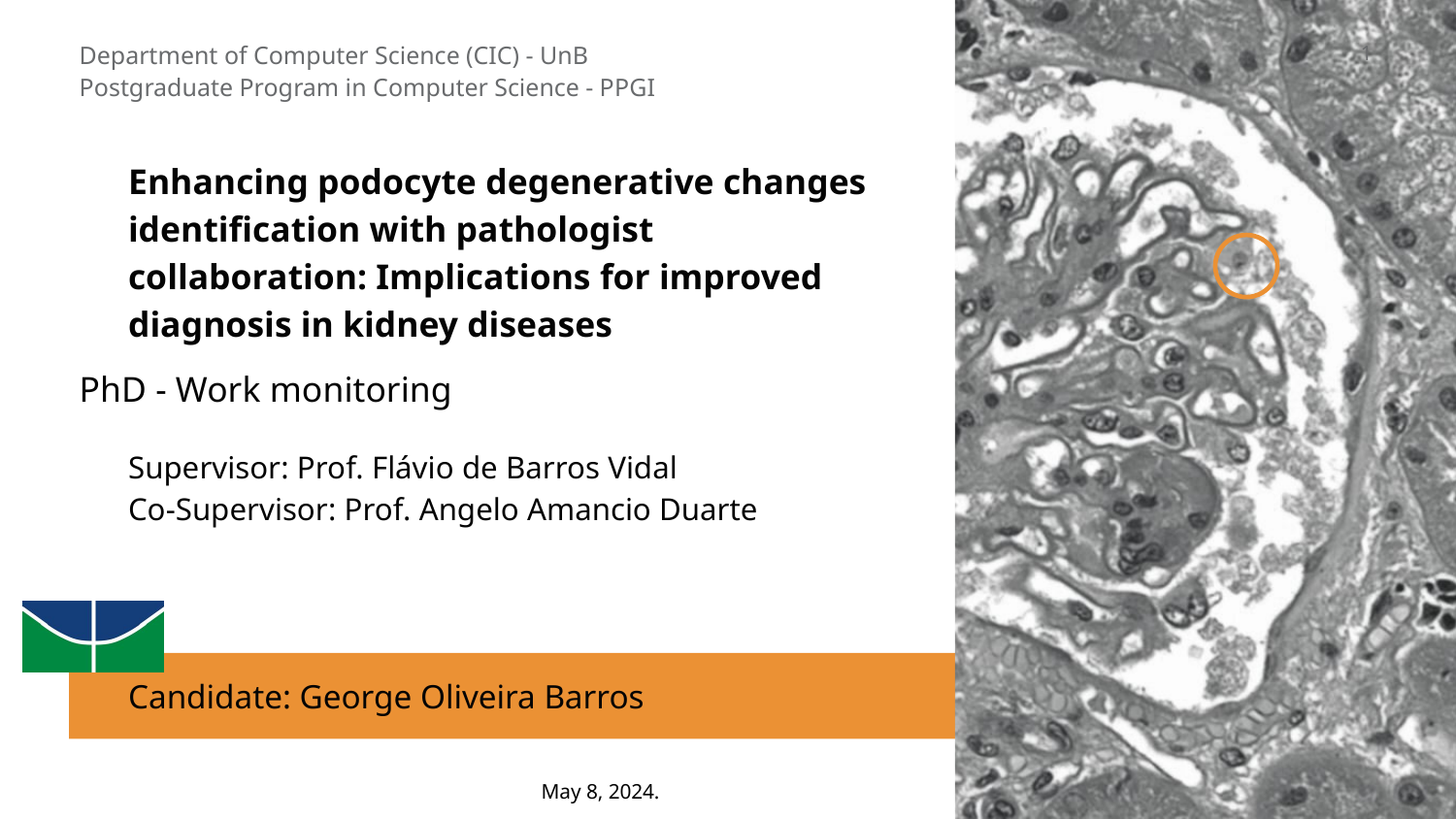

‹#›
Department of Computer Science (CIC) - UnB
Postgraduate Program in Computer Science - PPGI
# Enhancing podocyte degenerative changes identification with pathologist collaboration: Implications for improved diagnosis in kidney diseases
PhD - Work monitoring
Supervisor: Prof. Flávio de Barros Vidal
Co-Supervisor: Prof. Angelo Amancio Duarte
Candidate: George Oliveira Barros
May 8, 2024.

## Slide 2
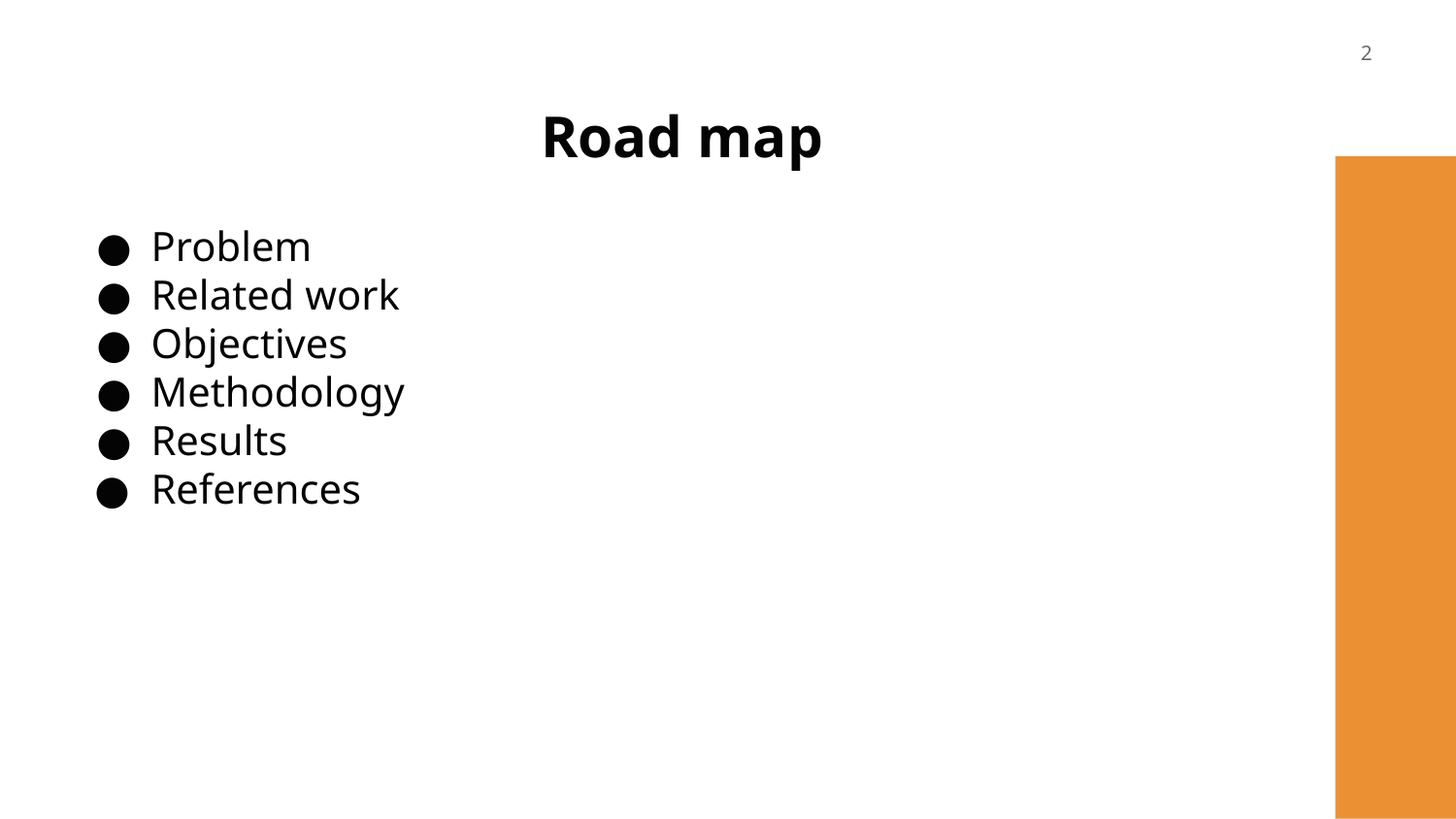

‹#›
# Road map
Problem
Related work
Objectives
Methodology
Results
References

## Slide 3
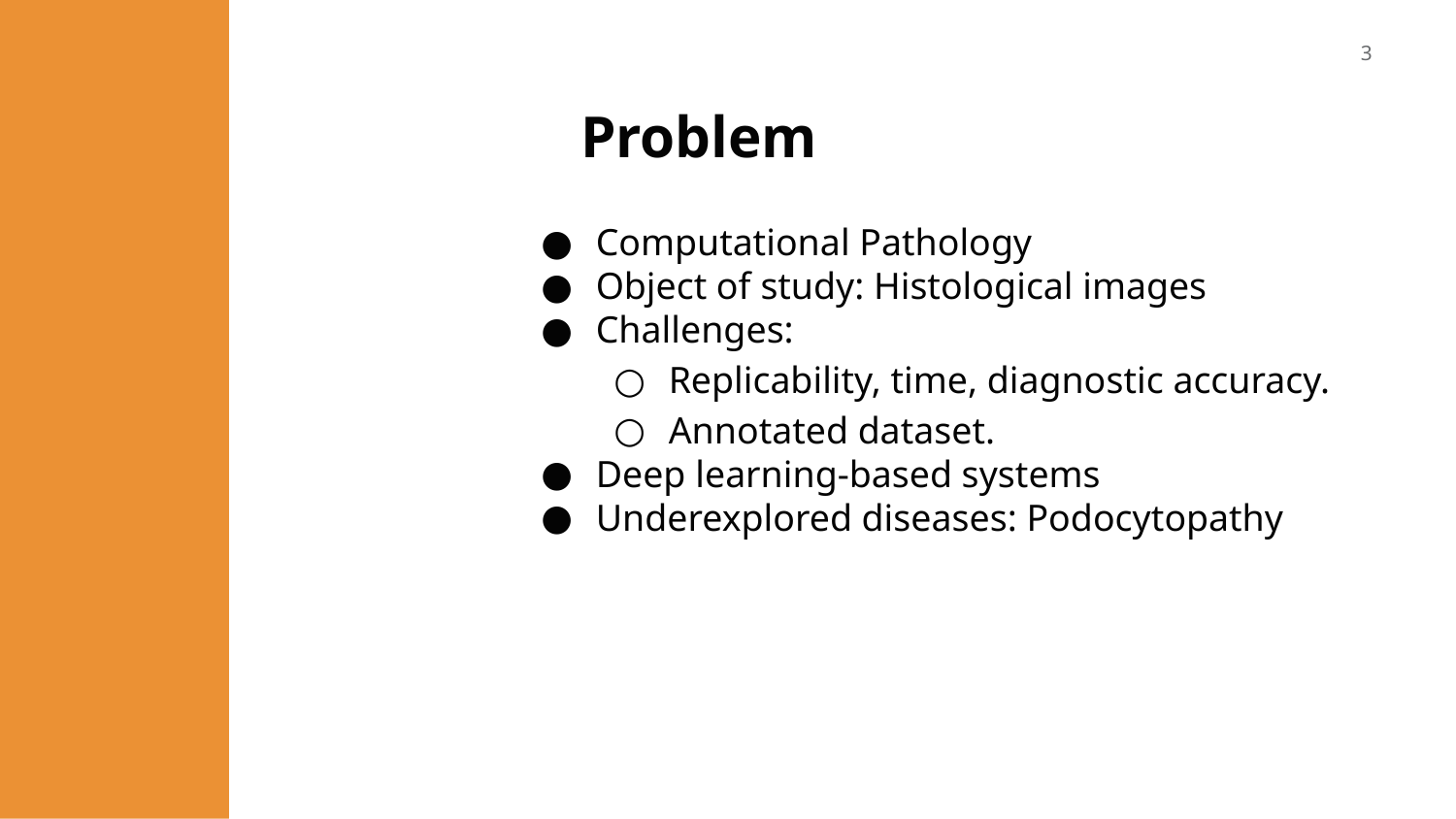

‹#›
# Problem
Computational Pathology
Object of study: Histological images
Challenges:
Replicability, time, diagnostic accuracy.
Annotated dataset.
Deep learning-based systems
Underexplored diseases: Podocytopathy

## Slide 4
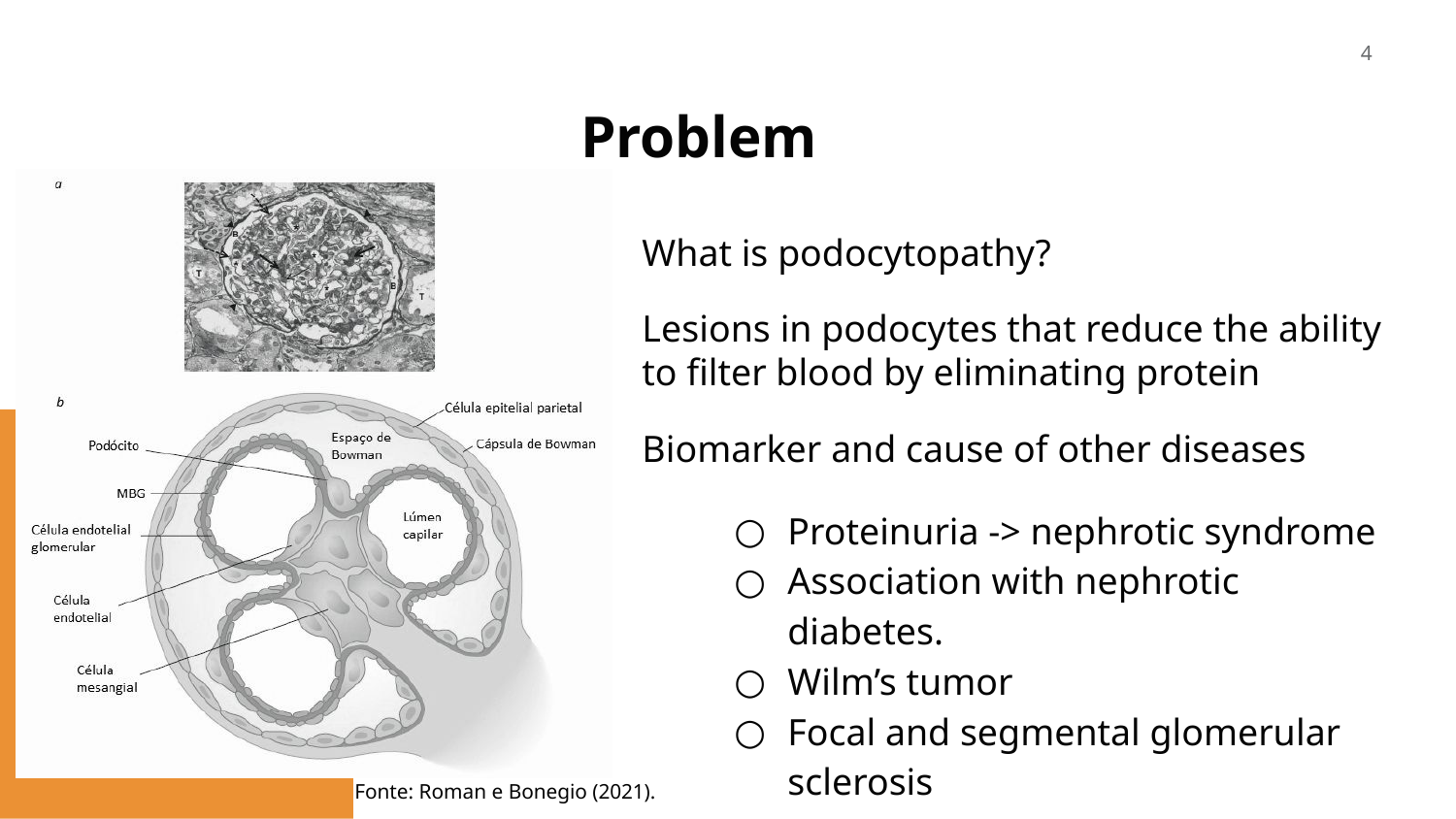

‹#›
# Problem
What is podocytopathy?
Lesions in podocytes that reduce the ability to filter blood by eliminating protein
Biomarker and cause of other diseases
Proteinuria -> nephrotic syndrome
Association with nephrotic diabetes.
Wilm’s tumor
Focal and segmental glomerular sclerosis
Fonte: Roman e Bonegio (2021).

## Slide 5
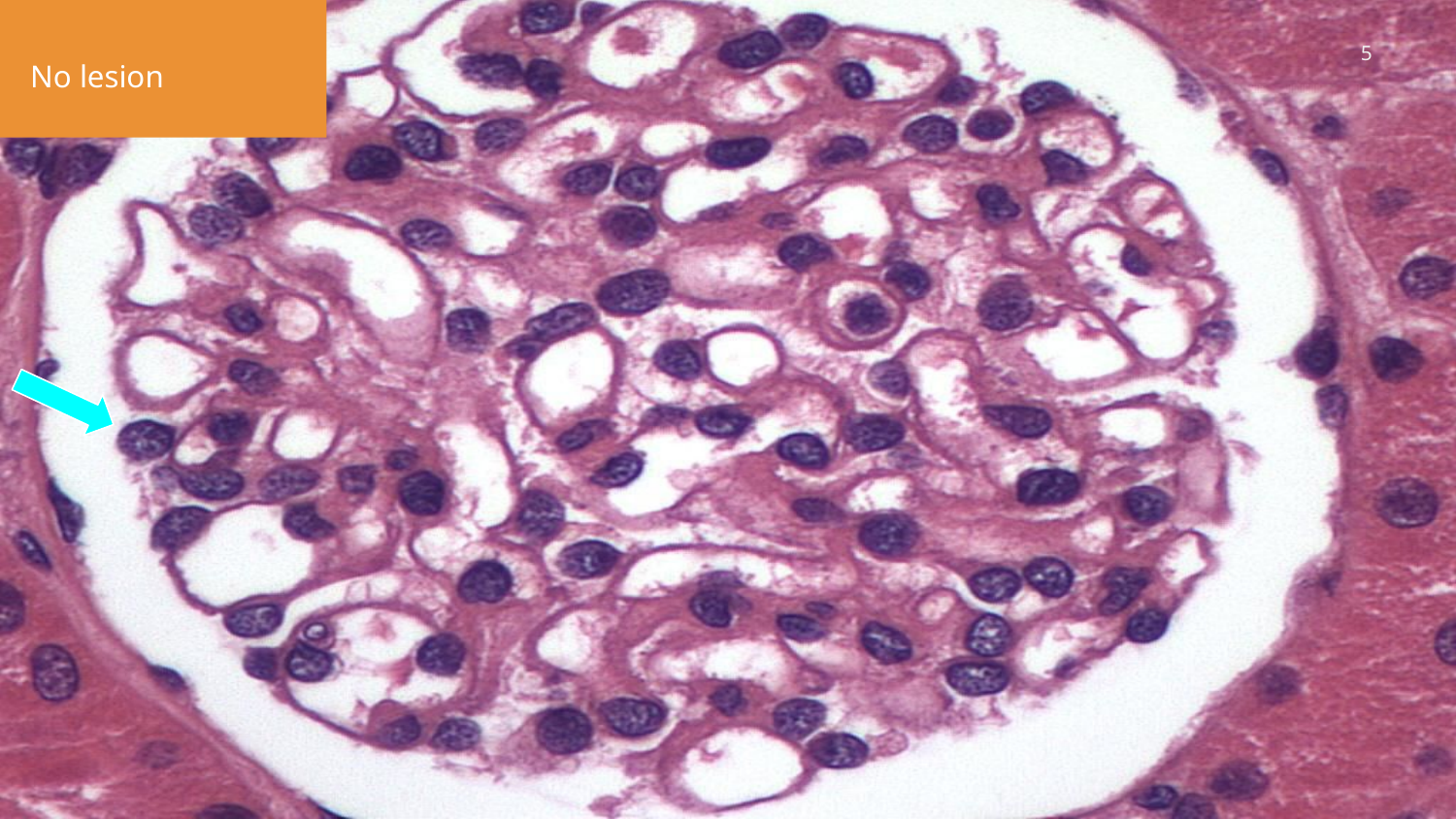

No lesion
‹#›

## Slide 6
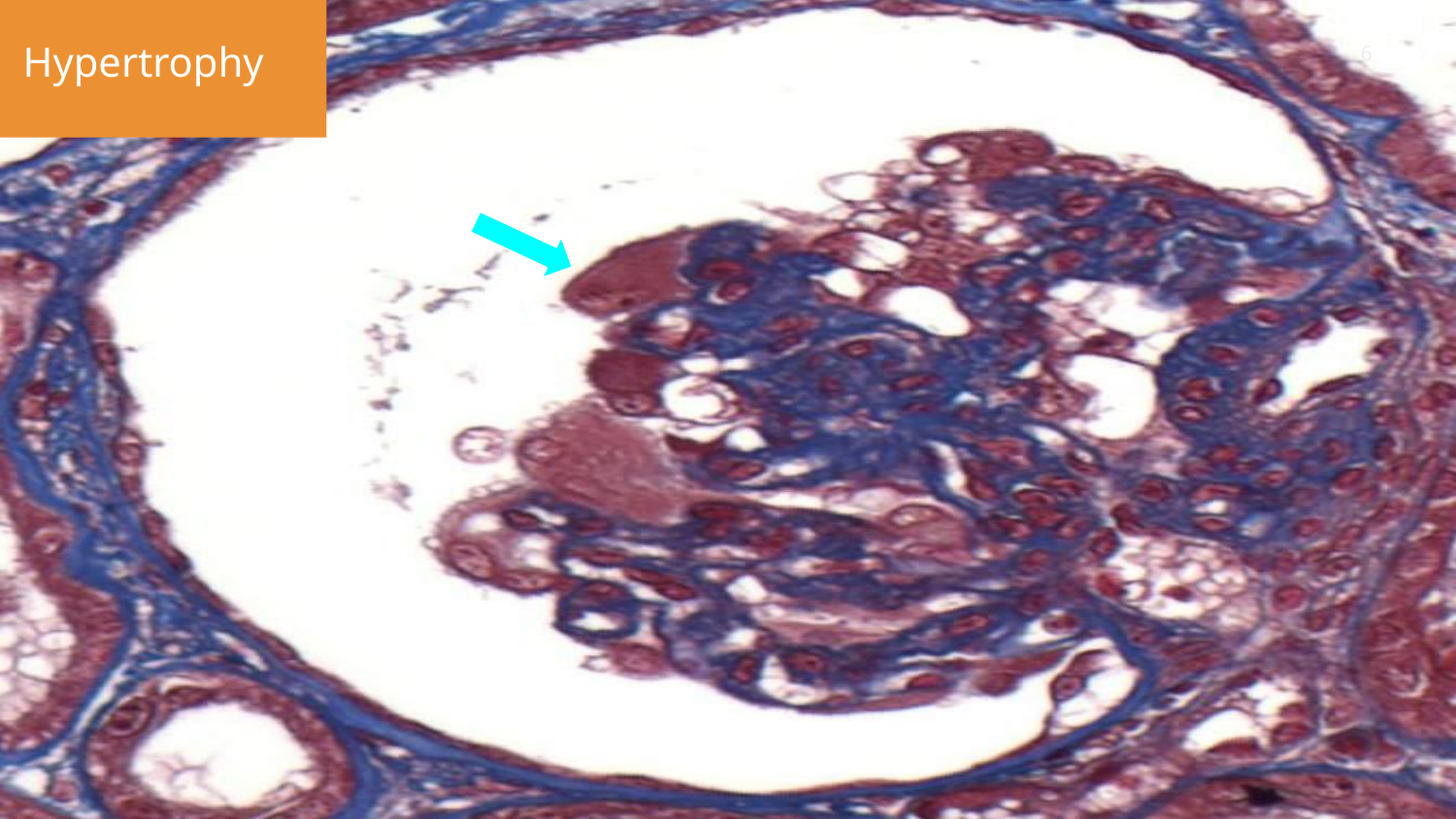

‹#›
Hypertrophy

## Slide 7
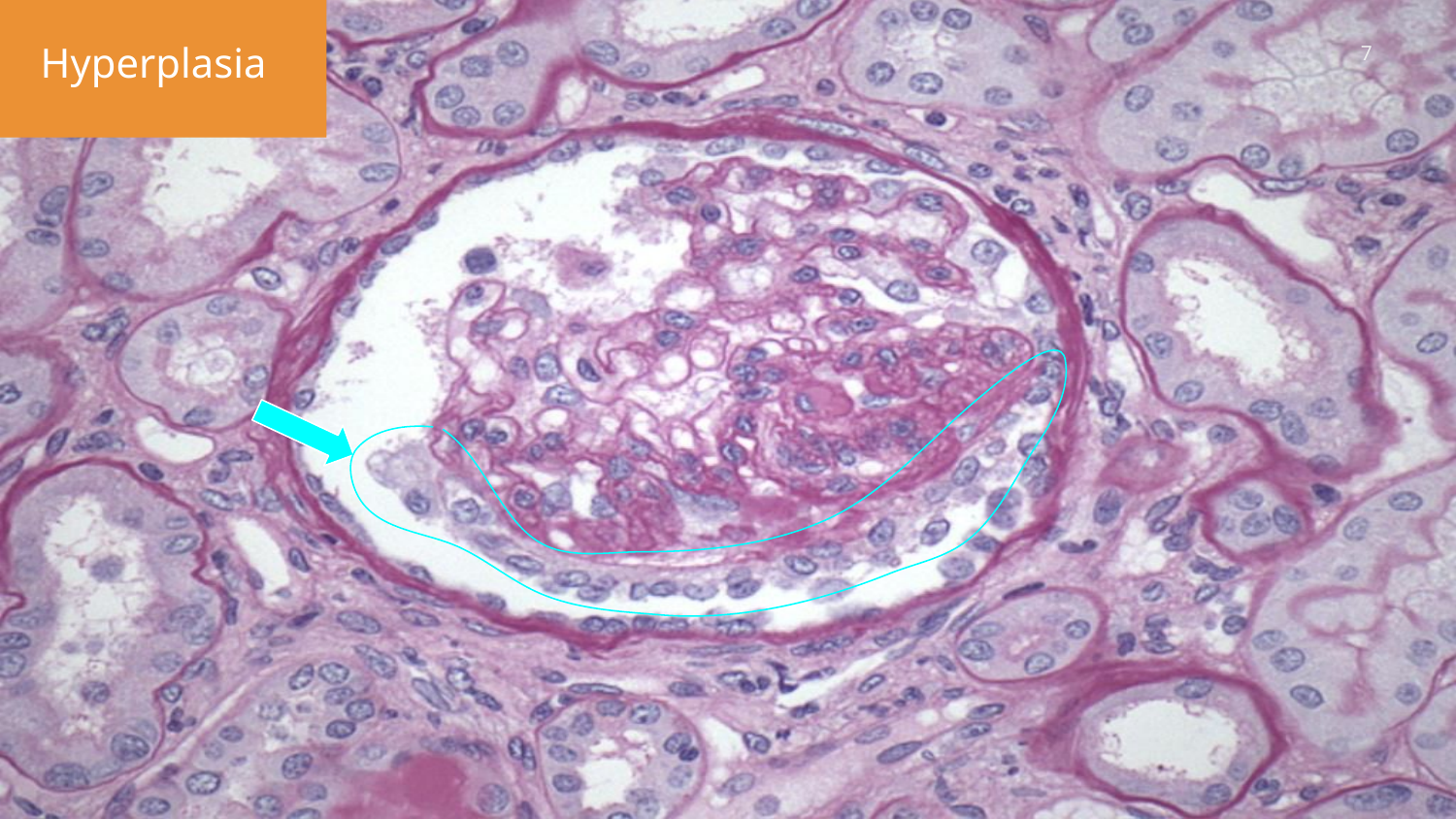

‹#›
Hyperplasia

## Slide 8
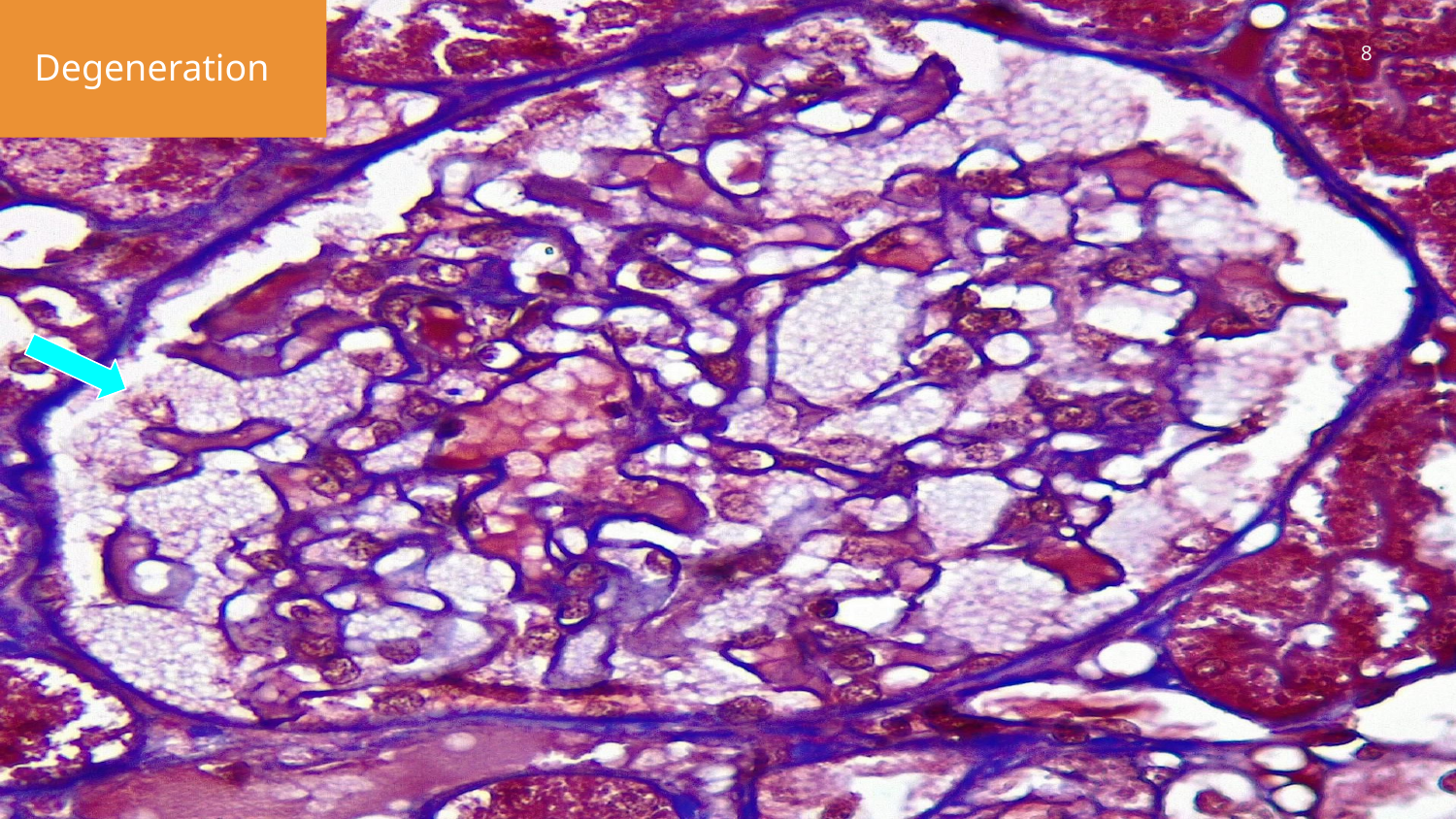

‹#›
Degeneration

## Slide 9
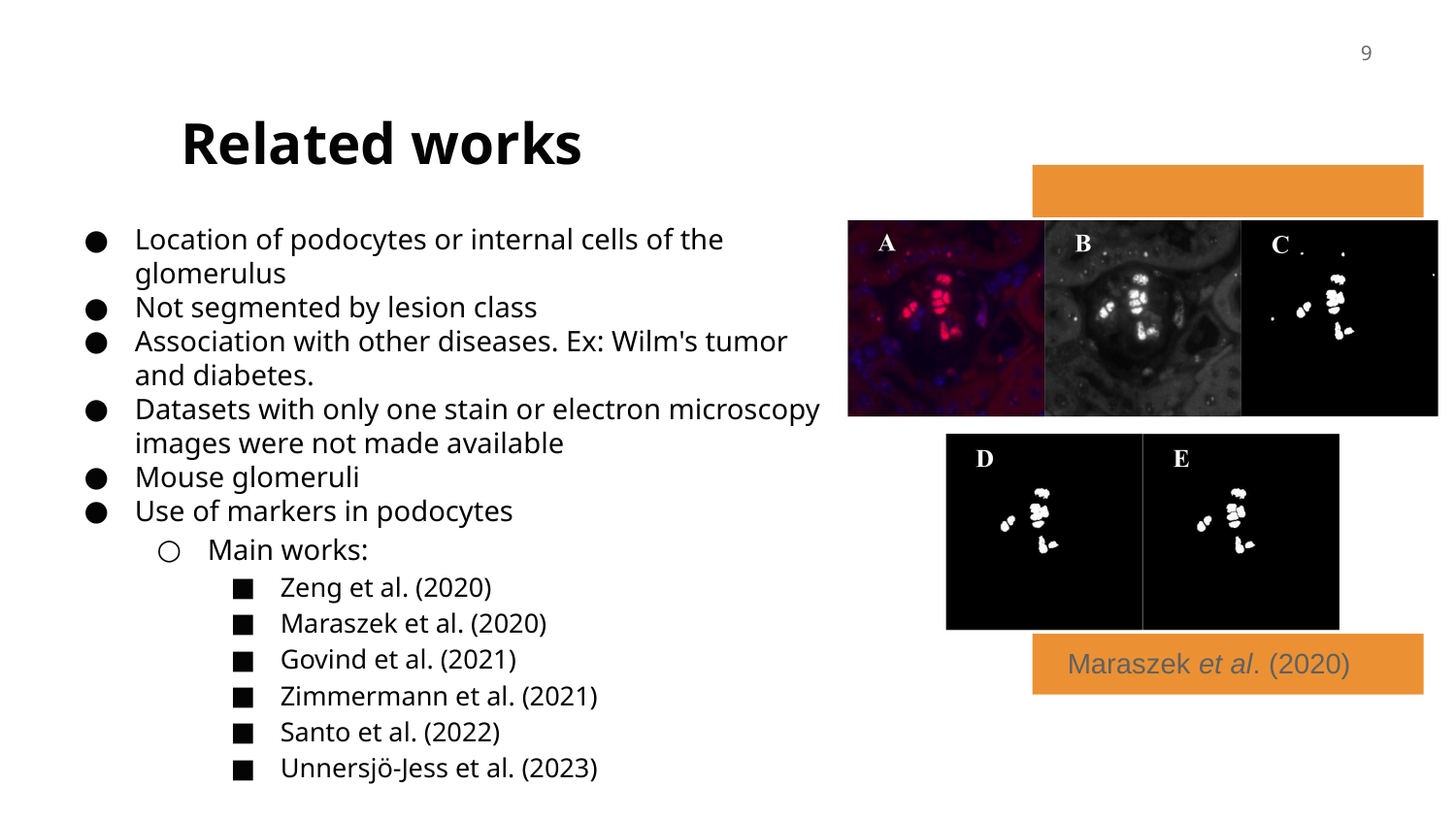

‹#›
# Related works
Location of podocytes or internal cells of the glomerulus
Not segmented by lesion class
Association with other diseases. Ex: Wilm's tumor and diabetes.
Datasets with only one stain or electron microscopy images were not made available
Mouse glomeruli
Use of markers in podocytes
Main works:
Zeng et al. (2020)
Maraszek et al. (2020)
Govind et al. (2021)
Zimmermann et al. (2021)
Santo et al. (2022)
Unnersjö-Jess et al. (2023)
Maraszek et al. (2020)

## Slide 10
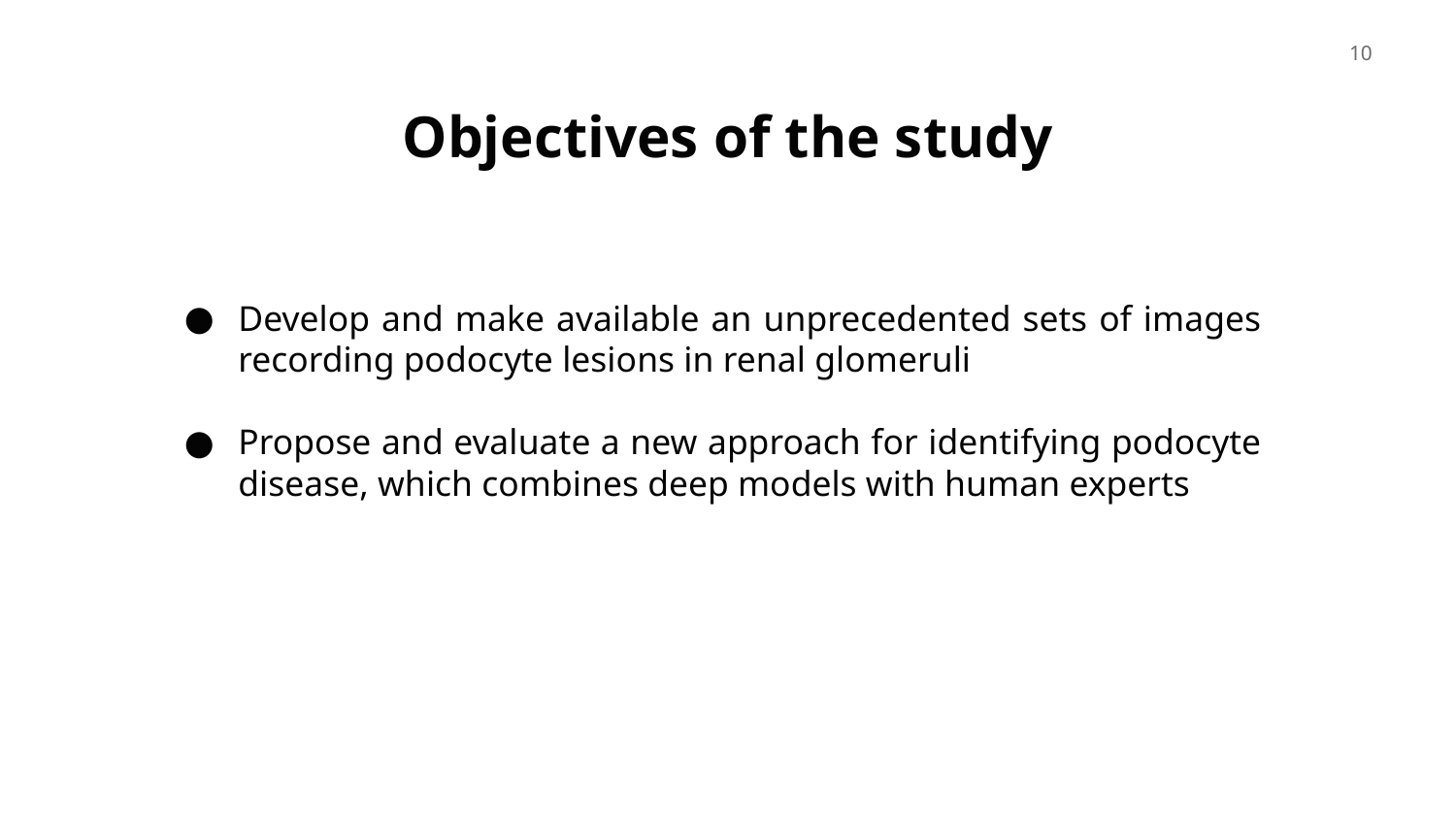

‹#›
Objectives of the study
Develop and make available an unprecedented sets of images recording podocyte lesions in renal glomeruli
Propose and evaluate a new approach for identifying podocyte disease, which combines deep models with human experts

## Slide 11
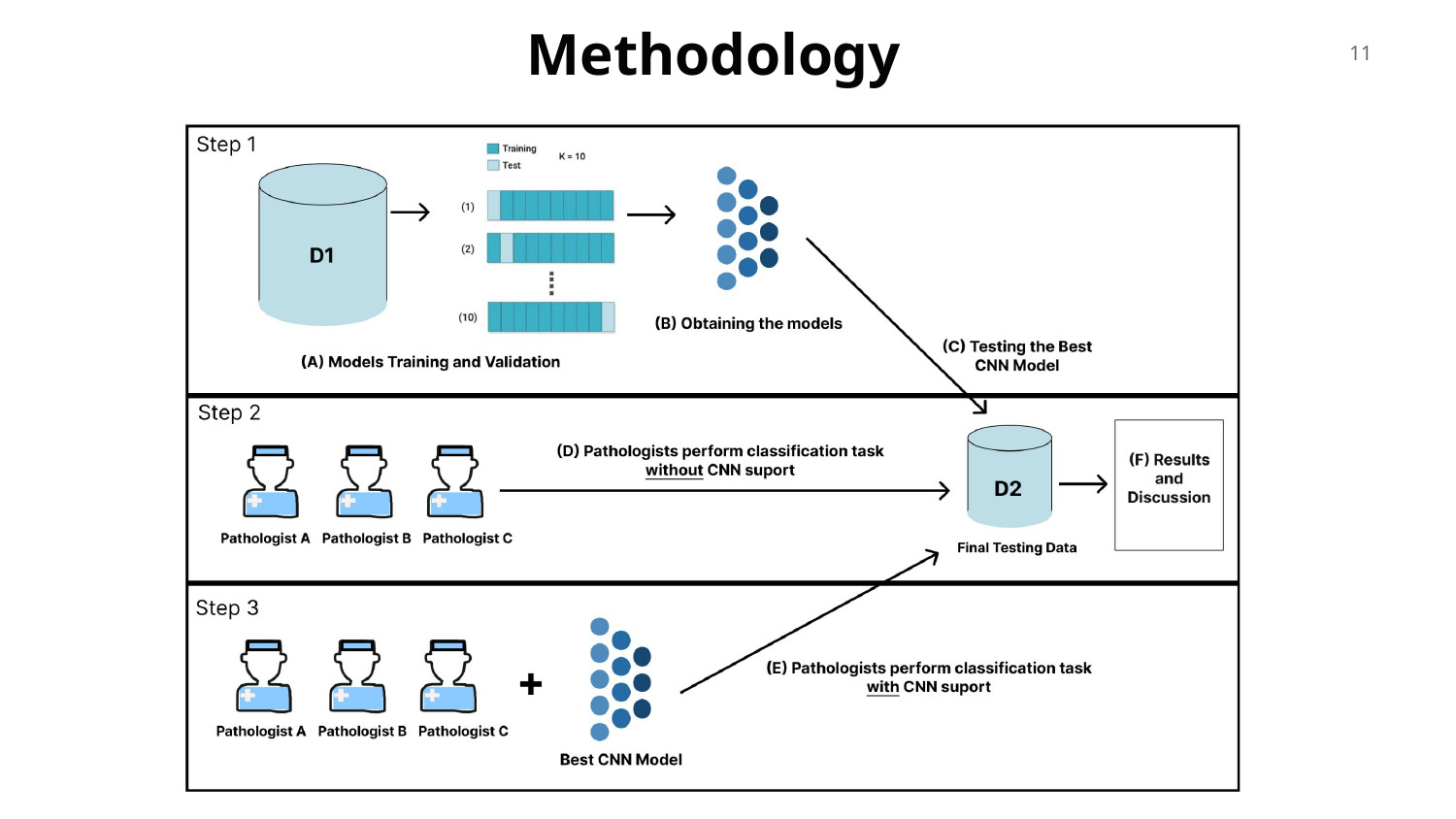

# Methodology
‹#›

## Slide 12
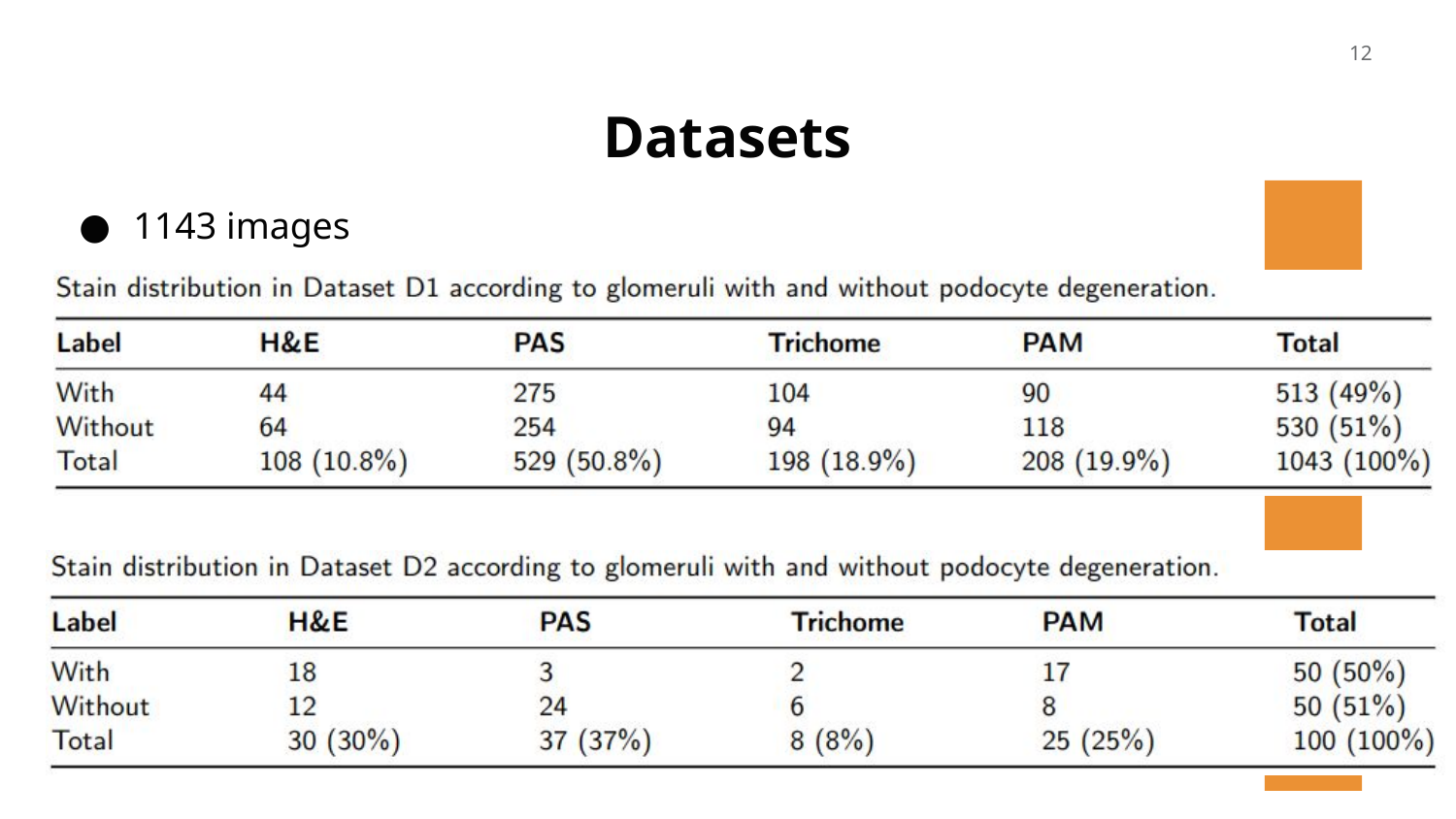

‹#›
# Datasets
1143 images

## Slide 13
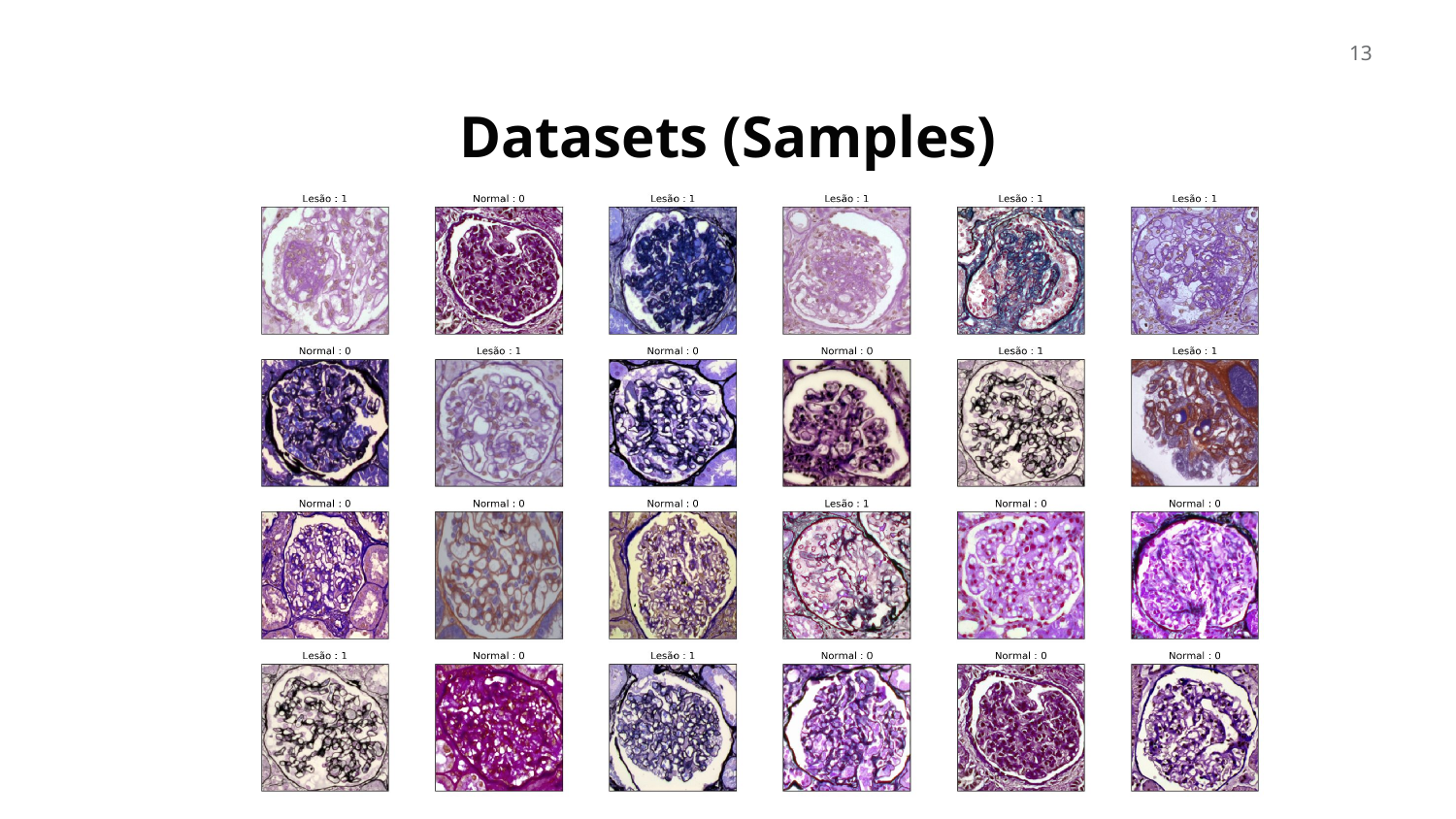

‹#›
# Datasets (Samples)

## Slide 14
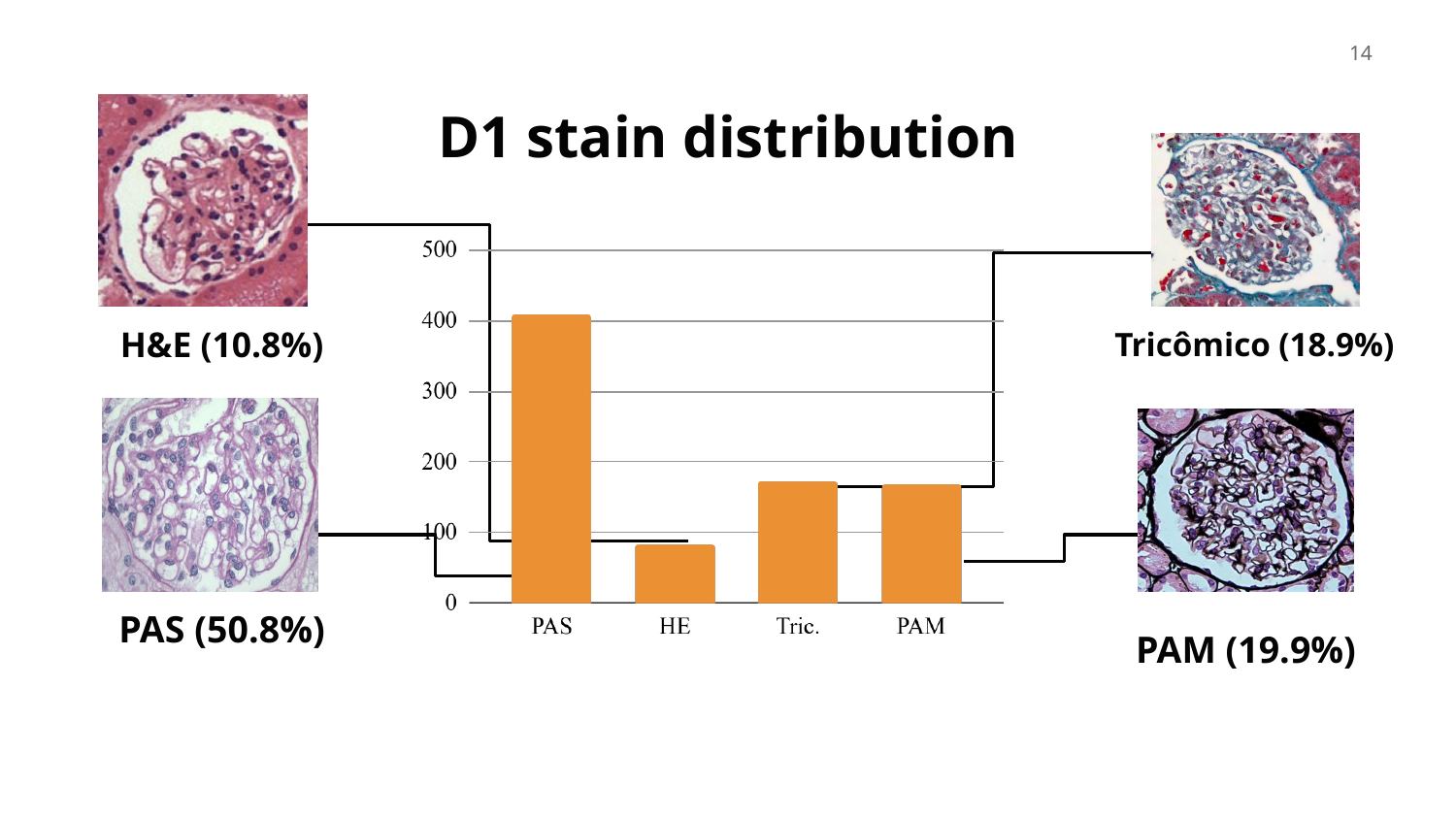

‹#›
# D1 stain distribution
10%
20%
H&E (10.8%)
Tricômico (18.9%)
49%
21%
PAM (19.9%)
PAS (50.8%)

## Slide 15
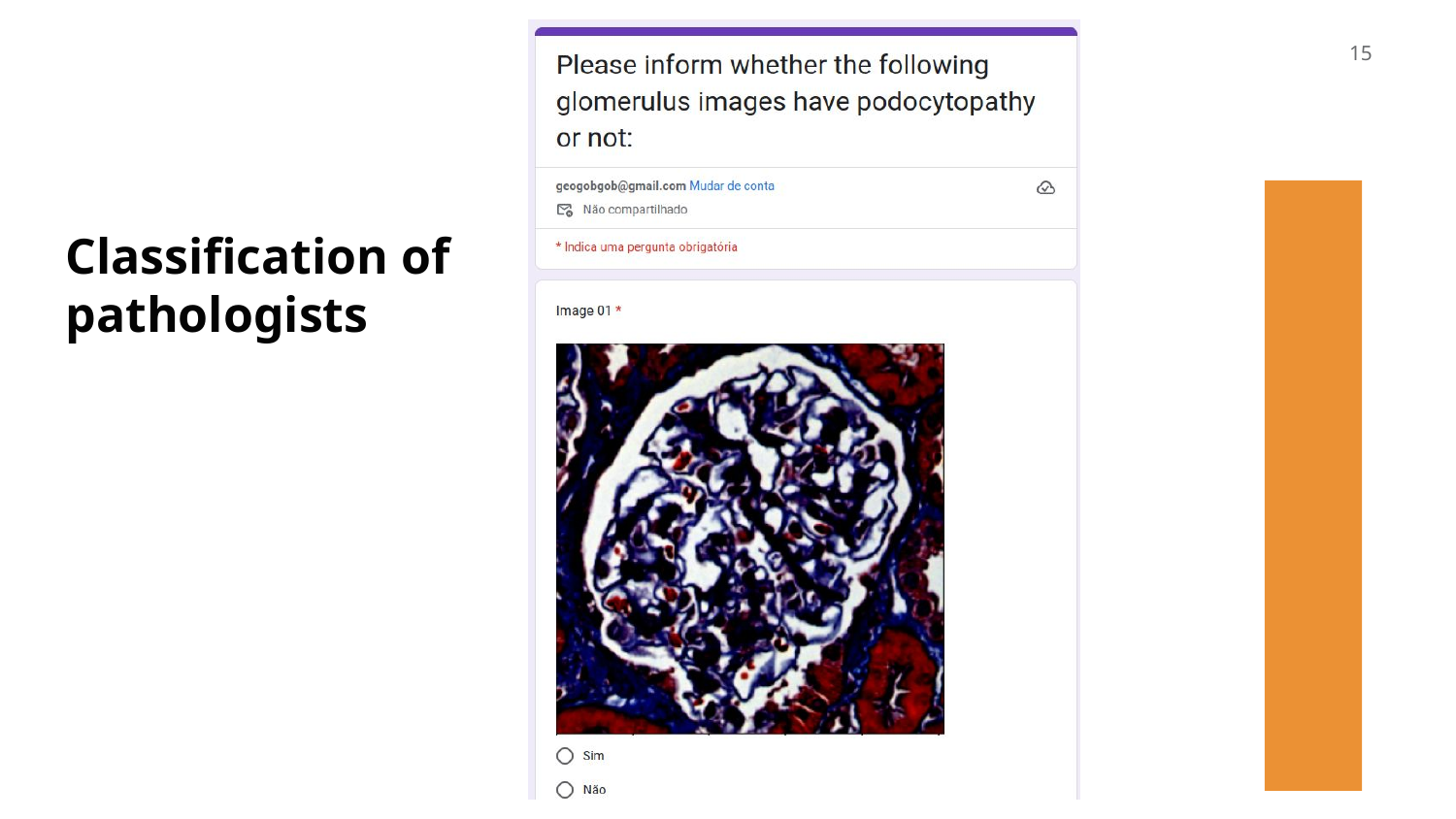

‹#›
# Classification of pathologists

## Slide 16
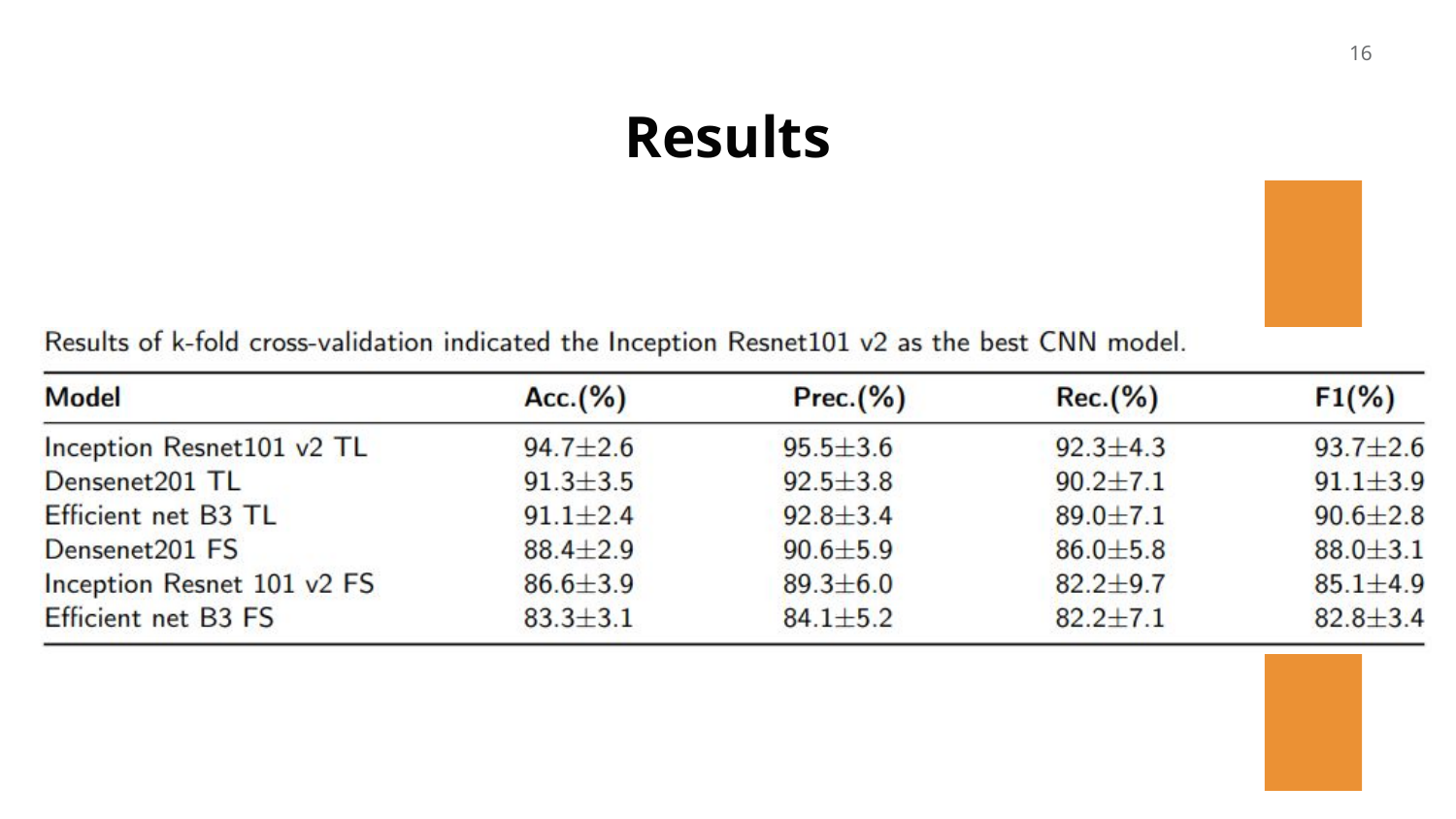

‹#›
# Results

## Slide 17
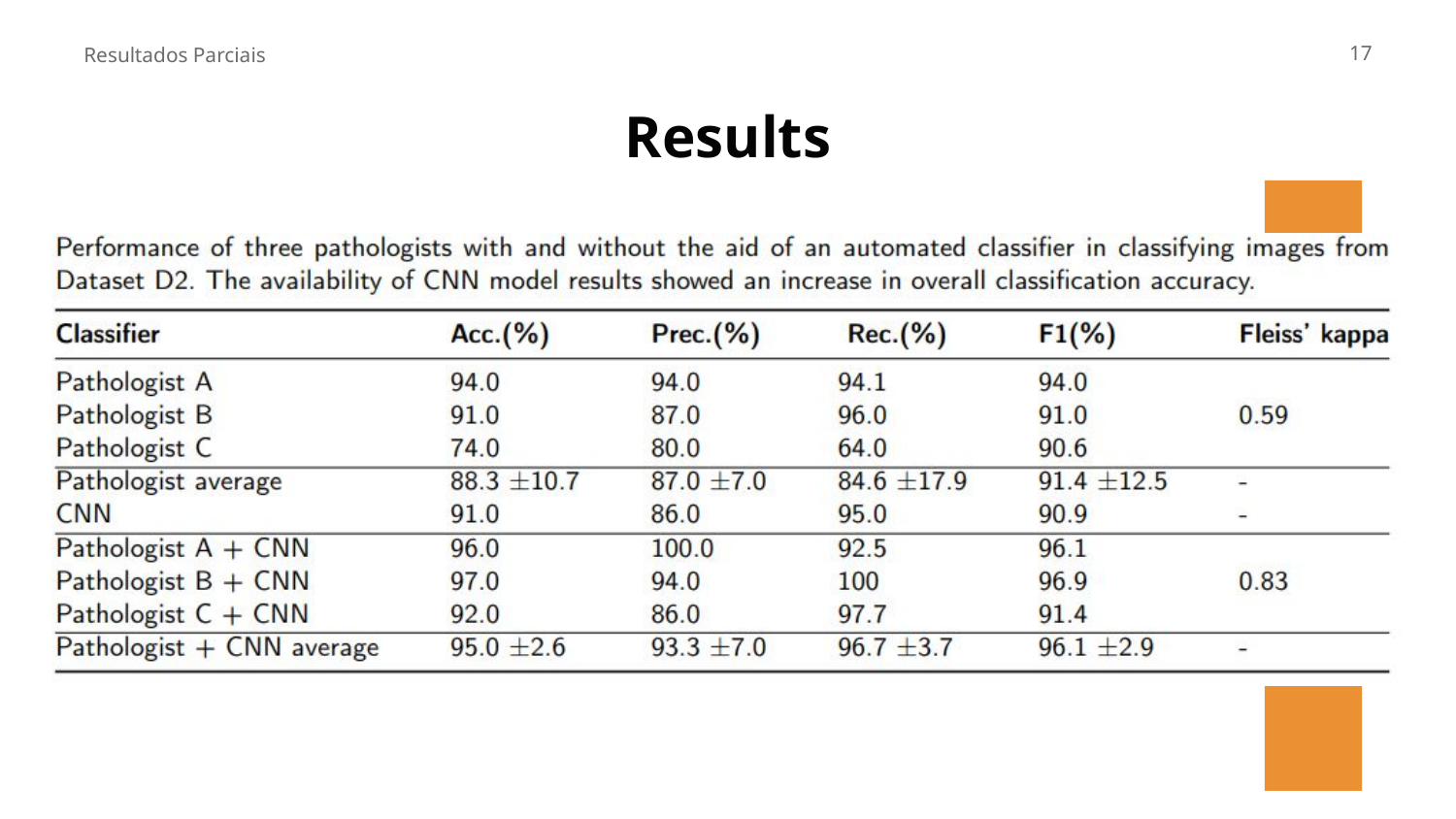

‹#›
Resultados Parciais
# Results

## Slide 18
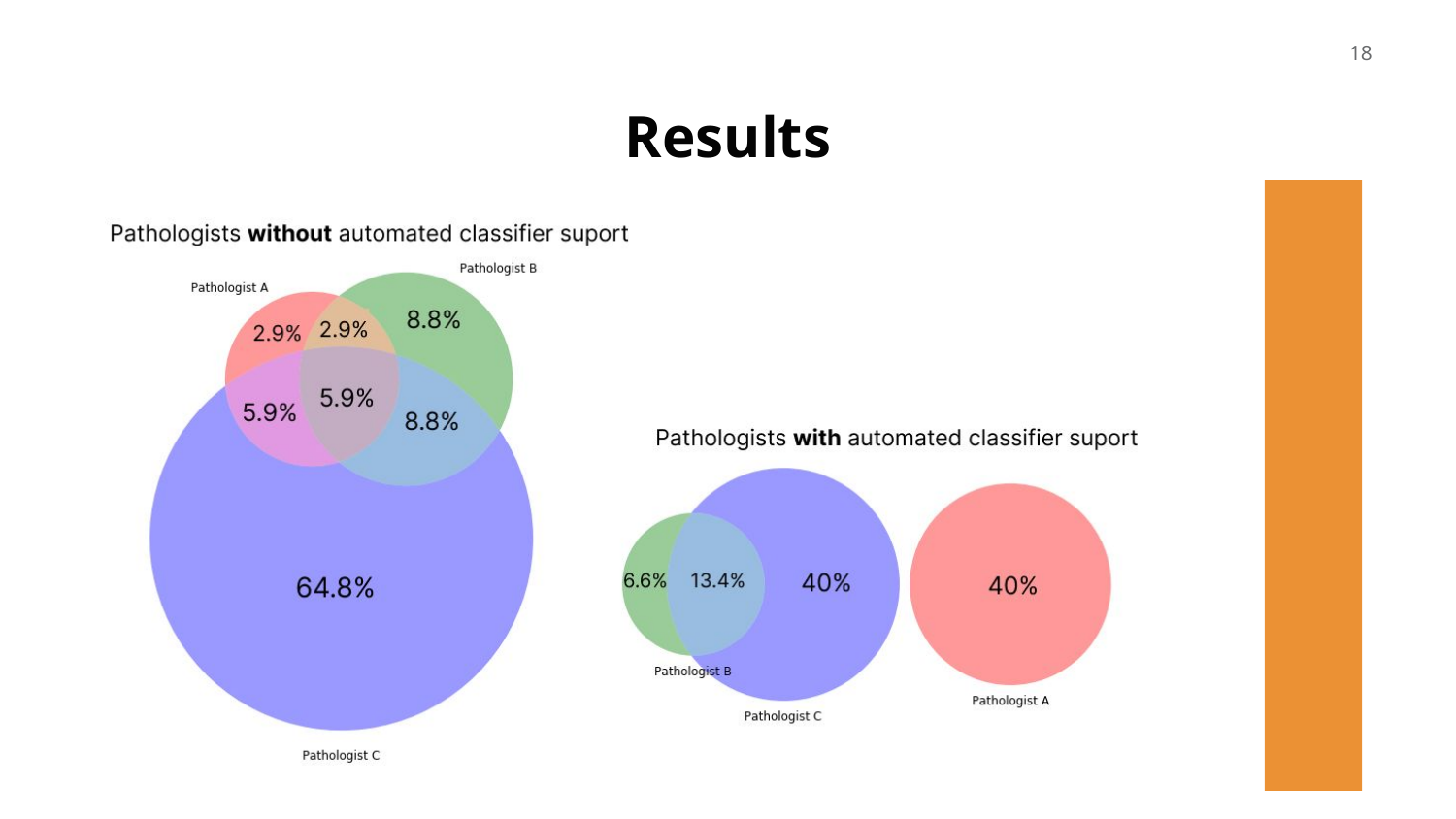

‹#›
# Results

## Slide 19
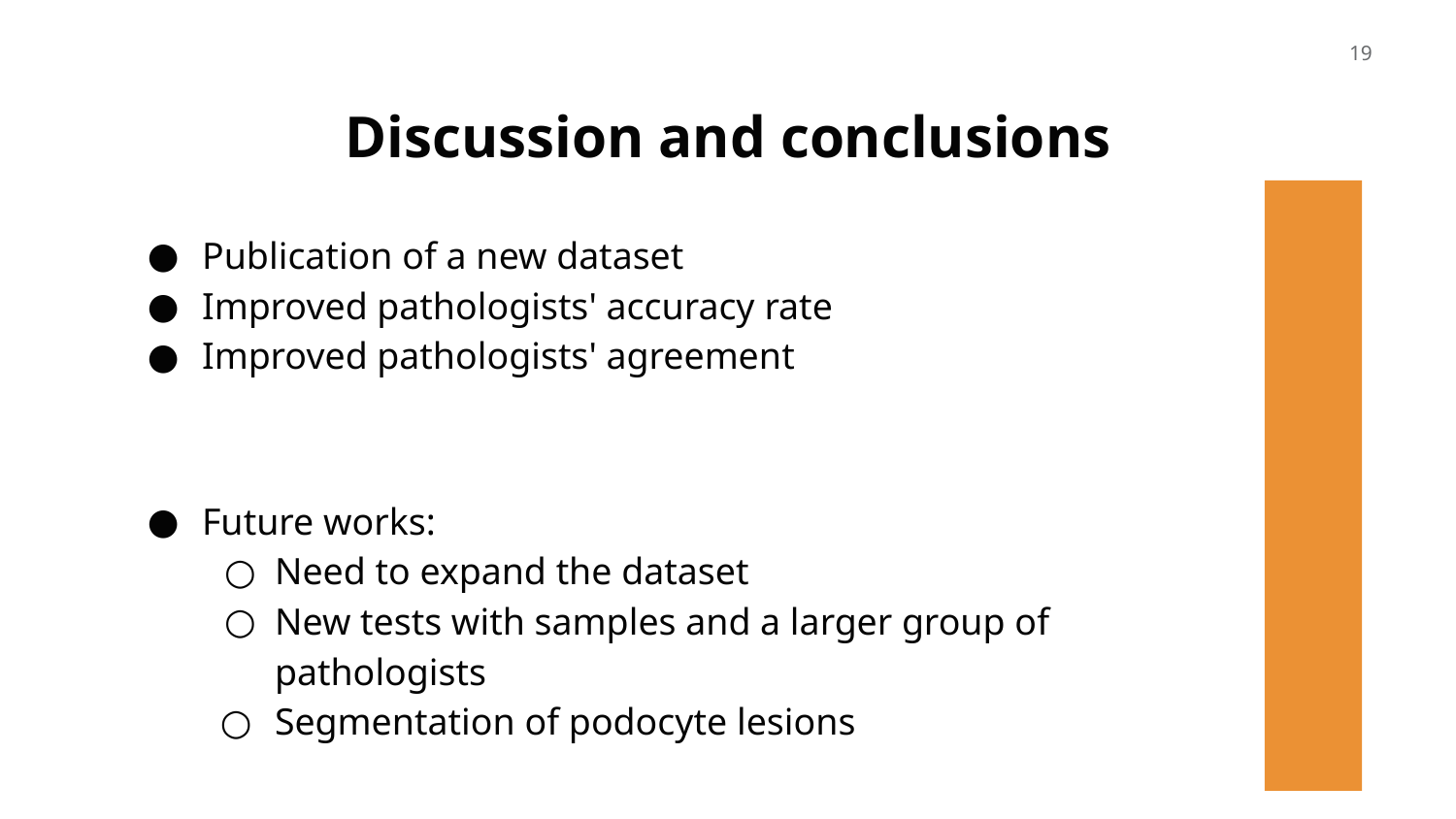

‹#›
# Discussion and conclusions
Publication of a new dataset
Improved pathologists' accuracy rate
Improved pathologists' agreement
Future works:
Need to expand the dataset
New tests with samples and a larger group of pathologists
Segmentation of podocyte lesions

## Slide 20
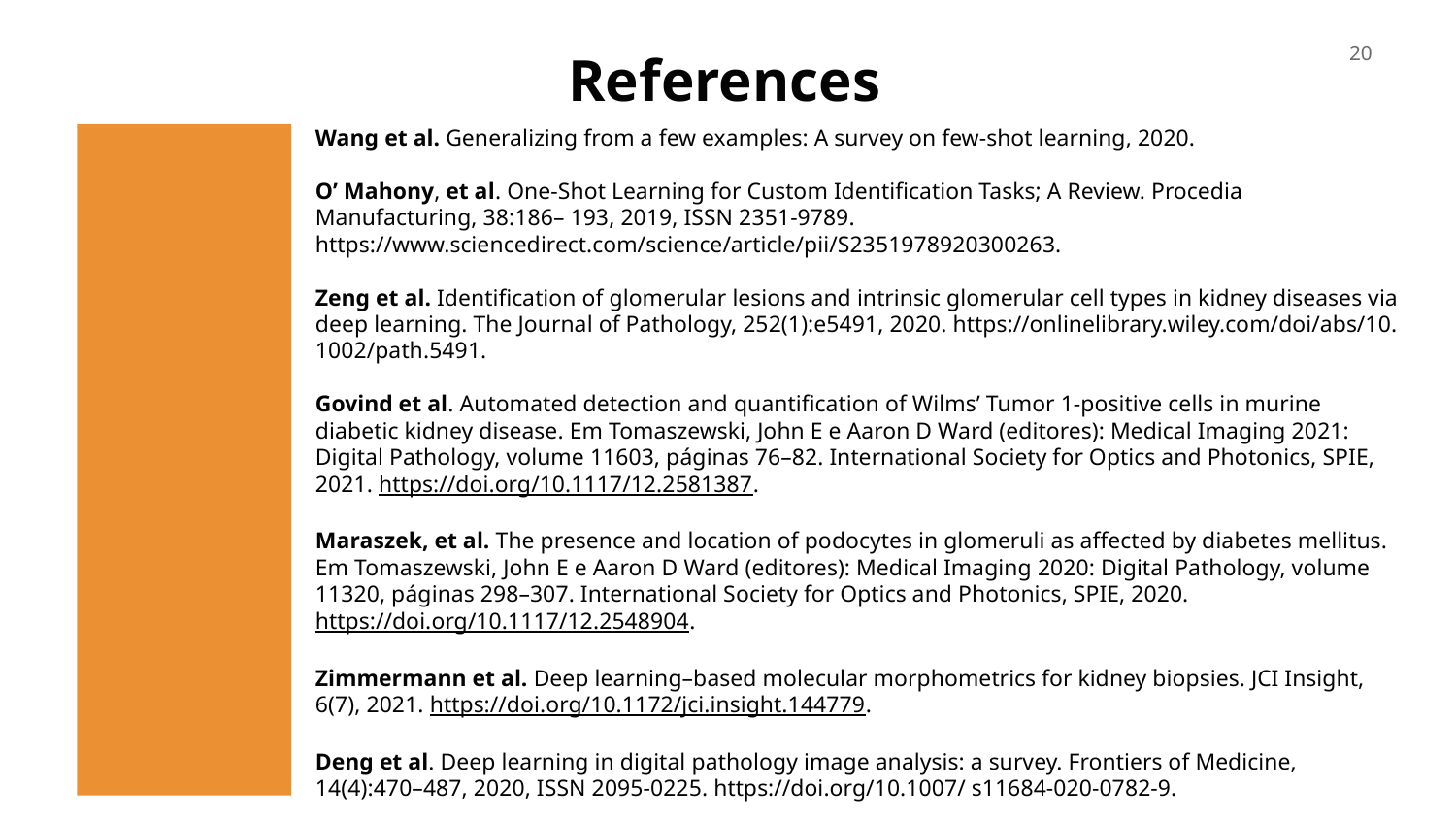

# References
‹#›
Wang et al. Generalizing from a few examples: A survey on few-shot learning, 2020.
O’ Mahony, et al. One-Shot Learning for Custom Identification Tasks; A Review. Procedia Manufacturing, 38:186– 193, 2019, ISSN 2351-9789. https://www.sciencedirect.com/science/article/pii/S2351978920300263.
Zeng et al. Identification of glomerular lesions and intrinsic glomerular cell types in kidney diseases via deep learning. The Journal of Pathology, 252(1):e5491, 2020. https://onlinelibrary.wiley.com/doi/abs/10. 1002/path.5491.
Govind et al. Automated detection and quantification of Wilms’ Tumor 1-positive cells in murine diabetic kidney disease. Em Tomaszewski, John E e Aaron D Ward (editores): Medical Imaging 2021: Digital Pathology, volume 11603, páginas 76–82. International Society for Optics and Photonics, SPIE, 2021. https://doi.org/10.1117/12.2581387.
Maraszek, et al. The presence and location of podocytes in glomeruli as affected by diabetes mellitus. Em Tomaszewski, John E e Aaron D Ward (editores): Medical Imaging 2020: Digital Pathology, volume 11320, páginas 298–307. International Society for Optics and Photonics, SPIE, 2020. https://doi.org/10.1117/12.2548904.
Zimmermann et al. Deep learning–based molecular morphometrics for kidney biopsies. JCI Insight, 6(7), 2021. https://doi.org/10.1172/jci.insight.144779.
Deng et al. Deep learning in digital pathology image analysis: a survey. Frontiers of Medicine, 14(4):470–487, 2020, ISSN 2095-0225. https://doi.org/10.1007/ s11684-020-0782-9.
